# Single-component near-infrared optogenetic systems for gene transcription regulation

**DOI:** 10.1038/s41467-021-24212-7

**Published:** 2021-06-23

**Authors:** Andrii A. Kaberniuk, Mikhail Baloban, Mikhail V. Monakhov, Daria M. Shcherbakova, Vladislav V. Verkhusha

**Affiliations:** 1 Department of Anatomy and Structural Biology, and Gruss-Lipper Biophotonics Center, Albert Einstein College of Medicine, Bronx, NY USA; 2 Medicum, Faculty of Medicine, University of Helsinki, Helsinki, Finland; 3 Science Center for Genetics and Life Sciences, Sirius University of Science and Technology, Sochi, Russia

**Keywords:** Optogenetics, Expression systems, Synthetic biology, Protein design

## Abstract

Near-infrared (NIR) optogenetic systems for transcription regulation are in high demand because NIR light exhibits low phototoxicity, low scattering, and allows combining with probes of visible range. However, available NIR optogenetic systems consist of several protein components of large size and multidomain structure. Here, we engineer single-component NIR systems consisting of evolved photosensory core module of *Idiomarina sp*. bacterial phytochrome, named iLight, which are smaller and packable in adeno-associated virus. We characterize iLight in vitro and in gene transcription repression in bacterial and gene transcription activation in mammalian cells. Bacterial iLight system shows 115-fold repression of protein production. Comparing to multi-component NIR systems, mammalian iLight system exhibits higher activation of 65-fold in cells and faster 6-fold activation in deep tissues of mice. Neurons transduced with viral-encoded iLight system exhibit 50-fold induction of fluorescent reporter. NIR light-induced neuronal expression of green-light-activatable CheRiff channelrhodopsin causes 20-fold increase of photocurrent and demonstrates efficient spectral multiplexing.

## Abstract (web summary)

Current near-IR optogenetic systems to regulate transcription consist of a number of large protein components. Here the authors report a smaller single-component near-IR system, iLight, developed from a bacterial phytochrome that they use to control gene transcription in bacterial and mammalian cells.

## Introduction

Light-induced protein–protein interactions exploited in the non-opsin optogenetic tools include homodimerization, heterodimerization, and oligomerization. Homodimerization of a small light–oxygen–voltage (LOV)-domain-containing protein, called VVD, is used for light-controlled transcription^1^. A LOV2 domain of phototropin 1 from *Avena sativa* and a modified PDZ domain are combined into an optogenetic system based on heterodimerization^2^. LOV2-based optogenetic tools enable light control of nuclear–cytoplasmic protein shuttling^3^. Cryptochrome 2 (CRY2) from *Arabidopsis thaliana* is another photoreceptor, which initially was applied with CIB1 partner in two-component heterodimerization approaches^4^. Later, its natural oligomerization ability was used in optogenetic clustering approaches^5^. Further tuning of the engineered light-activatable systems led to a design of the new generation of photodimerizers for advanced control of the protein localization^6^, cell signaling^7^, and recombinase activity^8^. All these optogenetic systems sense 440–480 nm light. Therefore, systems sensing light in a different spectral range are required for simultaneous use with blue-light-controlled optogenetic tools.

A class of photoreceptors called phytochromes stands apart from other photosensing proteins because of their ability to absorb far-red or near-infrared (NIR) light. All phytochromes utilize heme-derived linear tetrapyrrole compounds as their light-sensing chromophores. Red-light-triggered heterodimerization of a plant phytochrome B (PhyB) and a phytochrome-interacting factor 6 (PIF6) from *Arabidopsis* is successfully applied to transcriptional control^9^, cell signaling^10^, and protein localization^11^. Unlike plant phytochromes, which use phytochromobilin as a chromophore, a subclass of bacterial phytochrome photoreceptors (BphPs) incorporate biliverdin IXα (BV) tetrapyrrole^12^. As BV has the largest electron-conjugated system, it absorbs the most NIR-shifted light among all chromophores found in phytochromes. Moreover, in contrast to phytochromobilin or phycocyanobilin tetrapyrroles, BV is naturally present in all mammalian cells that makes BphPs the favorable templates to develop fluorescent proteins for applications in mammals^13,14^. BphPs exist in two interconvertible states, Pr (absorbs at 660–700 nm) and Pfr (absorbs at 740–780 nm). Upon NIR illumination, BphP-bound BV isomerizes via the fourth D-ring rotation around its 15–16 double bond. This *Z*–*E* isomerization results in the subsequent structural changes in an N-terminal photosensory core module (PCM) and an output (effector) domain of BphP. In turn, the PCM is formed by three domains, PAS (Per-ARNT-Sim), GAF (cGMP phosphodiesterase/adenylate cyclase/FhlA transcriptional activator), and PHY (phytochrome-specific), connected with α-helix linkers^15^.

Recently, the first optogenetic system that uses BphP from *Rhodopseudomonas palustris*, called RpBphP1, was developed. The NIR light-triggered heterodimerization of the full-length RpBphP1 with its natural RpPpsR2^16^ or engineered QPAS1^17^ binding partners allows precise control of gene transcription. BphP, serving as a light-sensing element of the RpBphP1–RpPpsR2 optogenetic system, belongs to non-canonical (bathy) BphPs, which in darkness adopt the Pfr state. Under NIR light of 740–780 nm, it undergoes the Pfr → Pr photoconversion, resulting in the reversible binding of RpPpsR2.

The substantial drawback of the currently available NIR optogenetic systems is the requirement to co-express two large protein components (i.e., PhyB phytochrome and PIF6 partner or RpBphP1 phytochrome and RpPpsR2 partner), meaning the need to co-transfect two plasmids or to co-transduce with two adeno-associated viruses (AAVs)^18^. Another drawback of the RpBphP1–RpPpsR2 system is its rather high background in darkness.

Here we aim to overcome the major drawbacks of the current NIR optogenetic systems by engineering optogenetic constructs based on modulation of the oligomeric state of a truncated canonical BphP (i.e., with the Pr ground state). We first chose wild-type canonical IsPadC (*Idiomarina* sp. *A28L* phytochrome-activated diguanylyl cyclase)^19^ BphP as a molecular template. We then reduce the size of IsPadC to its PCM, which is the minimal light-sensing module in phytochromes. We next evolve the IsPadC-PCM to engineer a chimeric gene transcription repressor in bacterial cells. We also engineer an IsPadC-PCM-based chimeric transcription factor for mammalian cells. We then validate the high efficiency of the light-induced gene transcription activation in cultured mammalian cells, primary isolated neurons, and intact mouse tissue in vivo. Lastly, we demonstrate the spectral multiplexing of the NIR light-controlled IsPadC-PCM-based optogenetic system with blue–green light-activatable tools, such as channelrhodopsin, and show the absence of their spectral crosstalk.

## Results

### Molecular evolution of IsPadC-PCM to light-controlled variant

**Fig. 1:**
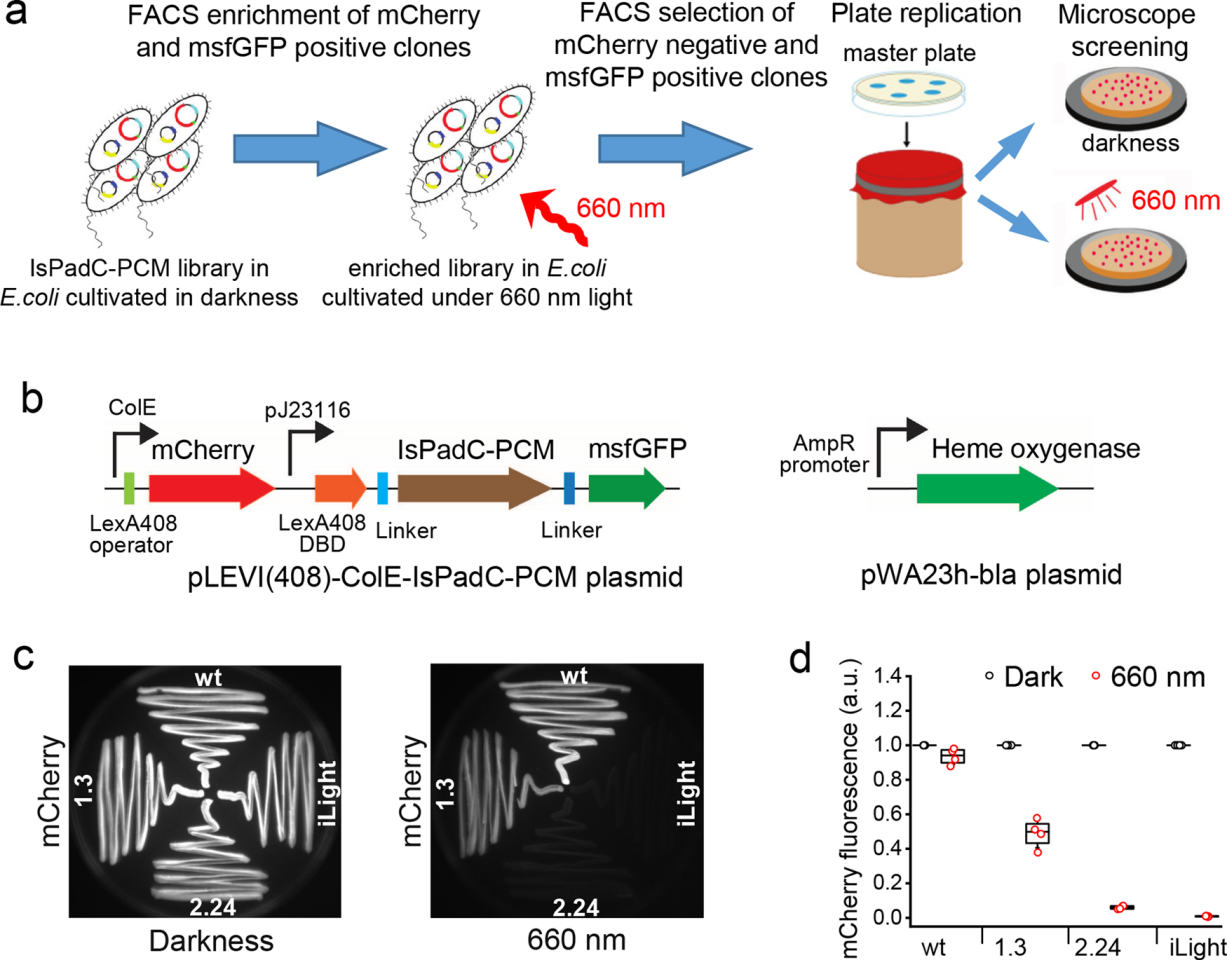
Molecular evolution of the IsPadC-PCM variants in bacteria. **a** Schematics outlining the high-throughput screening of IsPadC-PCM mutants with the light-controlled behavior. **b** Plasmids used in the molecular evolution of IsPadC-PCM. Left: pLEVI(408)-ColE-IsPadC-PCM encodes light-sensing protein LexA408-DBD-IsPadC-PCM-msfGFP under constitutive J23116 promoter and mCherry reporter under constitutive ColE promoter controlled by LexA408 operator. Right: pWA23h-bla encodes heme oxygenase for biliverdin production under constitutive β-lactamase promoter. **c** Repression of the mCherry reporter expression with 660 nm light. Streaks of bacteria expressing the indicated IsPadC-PCM mutants were grown on Petri dishes either in darkness or under 660 nm light and then imaged using 570/30 nm excitation and 615/40 nm emission filters. **d** Efficiency of the mCherry repression by selected IsPadC-PCM variants. mCherry signal was measured in bacterial suspensions grown in darkness or under 660 nm light. Box plots show the median (center line), first and third quartiles (box edges), 1× the SD (whiskers), and individual data points. *n* = 4 independent experiments. a.u., arbitrary units. Source Data are available as a Source Data file.

To avoid unwanted cyclase activity, we first removed the cyclase domain from wild-type IsPadC, resulting in its minimal PCM module. Next, to find an IsPadC-PCM mutant able to affect the level of reporter expression in bacteria, we performed molecular evolution (Fig. 1a) in which repression of the mCherry expression (reporter) was used as a readout (Fig. 1b). For this, we developed a high-throughput screening approach where NIR light-induced changes of an IsPadC-PCM oligomeric state were linked through a synthetic circuit to the expression of the mCherry reporter protein. The IsPadC-PCM was fused to a C terminus of the DNA-binding domain (DBD: amino acid residues 1–87)^20^ of LexA408-mutated repressor of the *Escherichia coli* SOS (coordinated response to DNA damage) regulon, which binds mutated operator and does not interfere with endogenous wild-type LexA protein and operator regions in bacterial SOS signaling pathway^21^.

Two low-copy plasmids, termed pWA23h-bla and pLEVI(408)-ColE-IsPadC-PCM, were co-transformed in TOP10 cells (Fig. 1b). The pWA23h-bla plasmid encoded heme oxygenase for BV synthesis in *E. coli*^22^ under control of a constitutively active weak β-lactamase promoter. The second plasmid encoded a light-sensitive repressor LexA408DBD-IsPadC-PCM-msfGFP under control of a weak constitutive promoter J23116 and the mCherry reporter under the control of a constitutive promoter ColE with the LexA408 operator located after the promoter. Changes of the IsPadC-PCM oligomeric state after photoconversion to the Pfr state should result in the formation of a functional dimer of the LexA408 DBDs. This dimer would then bind its cognate operator sequence placed after the constitutively active ColE promoter and repress the expression of mCherry.

To facilitate cell sorter selection of bacterial cells with the repressed mCherry expression, the C terminus of IsPadC-PCM was fused with a monomeric superfolder green fluorescent protein (msfGFP) protein, allowing selection of the cells with full-length IsPadC-PCM. Moreover, the msfGFP signal can be used to normalize the mCherry signal during the screening of clones from the colony replicas (Supplementary Fig. 1).

Libraries of the random IsPadC-PCM mutants in bacterial cells were grown overnight in darkness and enriched for mCherry- and msfGFP-positive cells using a cell sorter. The enriched library was then grown overnight under 660 nm light and the msfGFP-positive and mCherry-negative cells were collected (Supplementary Fig. 2). To further screen the collected cells on Petri dishes, the bacterial clones were replicated and cultivated in darkness and under 660 nm light. After the initial screening, the clones with the highest ratio of the mCherry repression were characterized in detail (Supplementary Fig. 3).

As a result, after the first round of mutagenesis, a clone 1.3 with 2-fold decrease of the mCherry signal was selected. After the next two rounds of random mutagenesis, we obtained an IsPadC-PCM variant having 9 amino acid substitutions (Supplementary Table 1) and resulting in ~115-fold repression of the ColE-driven mCherry reporter expression (Fig. 1c, d and Supplementary Fig. 4). This variant was named iLight (Supplementary Fig. 5).

### iLight-based optogenetic system for repression of protein production

**Fig. 2:**
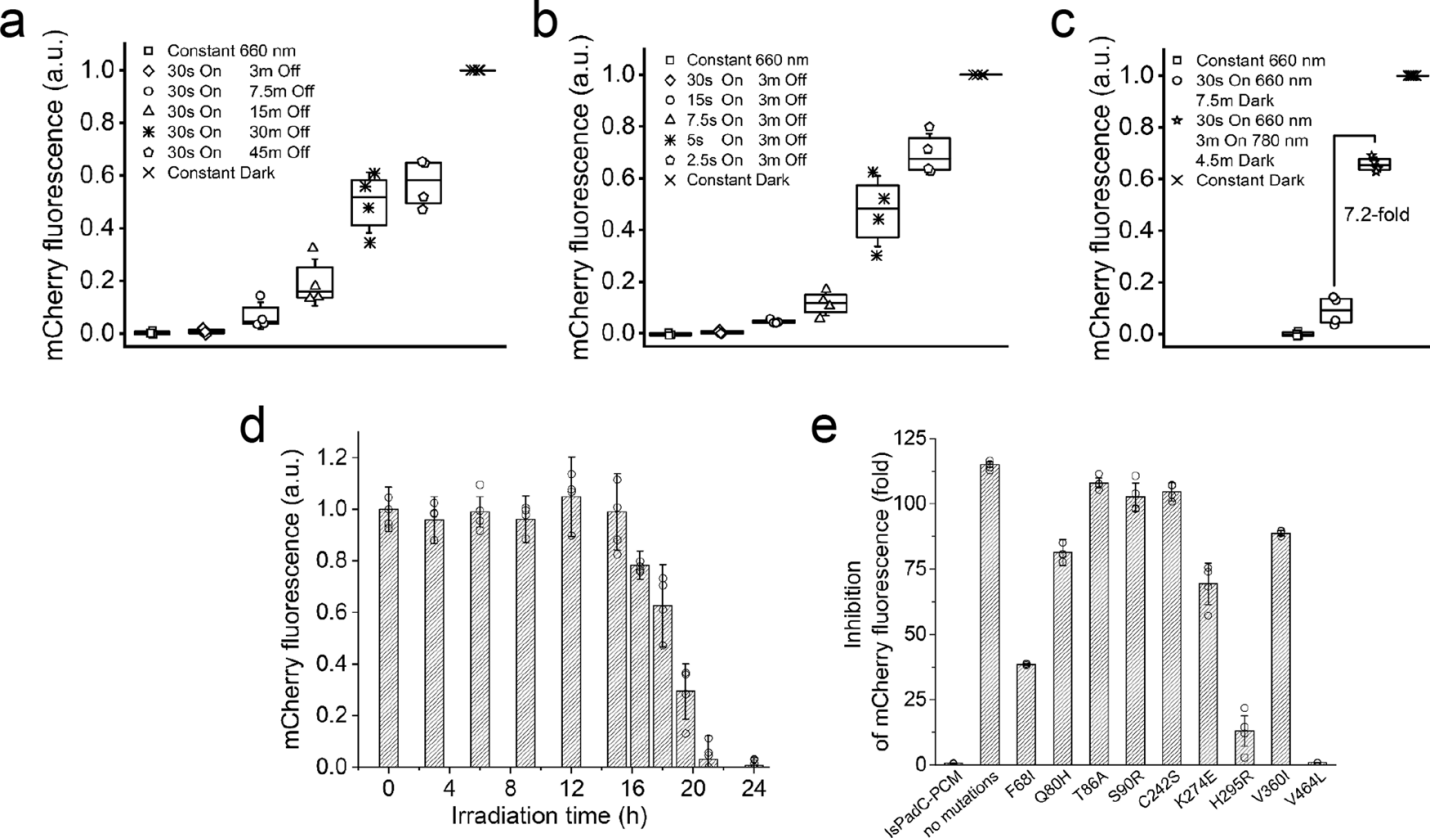
iLight-based system for the light-induced repression of protein expression in bacteria. **a**, **b** Dependence of the inhibition efficiency on the duration of (**a**) Off and (**b**) On time of the 660 nm illumination. **c** Effect of 780 nm light, which photoconverts iLight from the Pfr state to the Pr state, on the inhibition of the mCherry reporter production. Box plots show the median (center line), first and third quartiles (box edges), 1× the SD (whiskers), and individual data points. The illumination steps described in **a**–**c** were repeated in the loop and the total duration of each of these experiments was 16 h. **d** Inhibition of the mCherry reporter expression in the ongoing mCherry expression conditions. Bacterial samples expressing the mCherry reporter were cultured in darkness and then transferred to 660 nm light for the indicated illumination time periods, after which the fluorescence intensity of the bacteria was analyzed. Each bacterial sample was cultured for total 24 h before the analysis. Error bars, SD (*n* = 4 independent experiments). **e** Efficiency of the inhibition of the mCherry reporter production by various single-point mutants of iLight. Error bars, SD (*n* = 4 independent experiments). In **a**–**d**, a.u., arbitrary units. Source Data are available as a Source Data file.

To determine the optimal illumination regime of the iLight-based repression system in bacterial cells, we next studied different illumination conditions (Fig. 2a–c). We assembled a multichannel 660 nm light emitting diode (LED) array (Supplementary Fig. 6) to illuminate bacteria grown in 15 ml tubes. The LED array was based on Arduino microprocessor programmed to study the dependence of the repression efficiency on the duration of Off (Fig. 2a) and On (Fig. 2b) time of 660 nm illumination. These scripts control both the On/Off illumination cycle and the light power for each tube.

The bacterial iLight optogenetic system enabled the fine-tuning of the mCherry protein repression by varying of 660 nm On/Off illumination cycle. Fifteen seconds of 660 nm illumination sufficed to repress mCherry expression with a 115-fold contrast (Fig. 2a, b). Furthermore, the repression could be switched off by iLight photoconversion back from the Pfr state to the Pr state with 780 nm light. When the illumination cycle consisted of 30 s of 660 nm light, followed by 3 min of 780 nm light and 4.5 min in darkness, bacteria restored up to 75% of the mCherry expression (Fig. 2c). Notably, in the dark, the expression level of the mCherry reporter did not depend on whether the iLight system was co-expressed or not (Supplementary Fig. 7).

We next tested whether the iLight system is able to repress the gene expression when it is ongoing. For this, we cultured bacteria for a total 24 h, with various darkness and subsequent 660 nm illumination periods. Repression of the mCherry expression was observed for the darkness periods up to ~8 h, which were followed by the iLight activation (Fig. 2d). The mCherry repression reached 50% for 5.5 h of ongoing expression followed by 18.5 h of the 660 nm illumination.

To determine the contribution of each of nine amino acid substitutions found in iLight (Supplementary Table 1) on its gene suppression activity, we sequentially reverted them to those in wild-type IsPadC-PCM and determined the efficiency of the resulting single-point iLight mutants on the repression of the gene expression (Fig. 2e). Each of the Q80H, T86A, S90R, C242S, K274R, and V360I single-point mutations had a minor effect on the inhibition performance of the bacterial iLight system. In contrast, the F68I, H295R, and V464L amino acid substitutions significantly affected the iLight performance, with the V464L substitution lowering the inhibition efficiency of the gene expression to only 1.6-fold.

### Characterization of the purified iLight variant

**Fig. 3:**
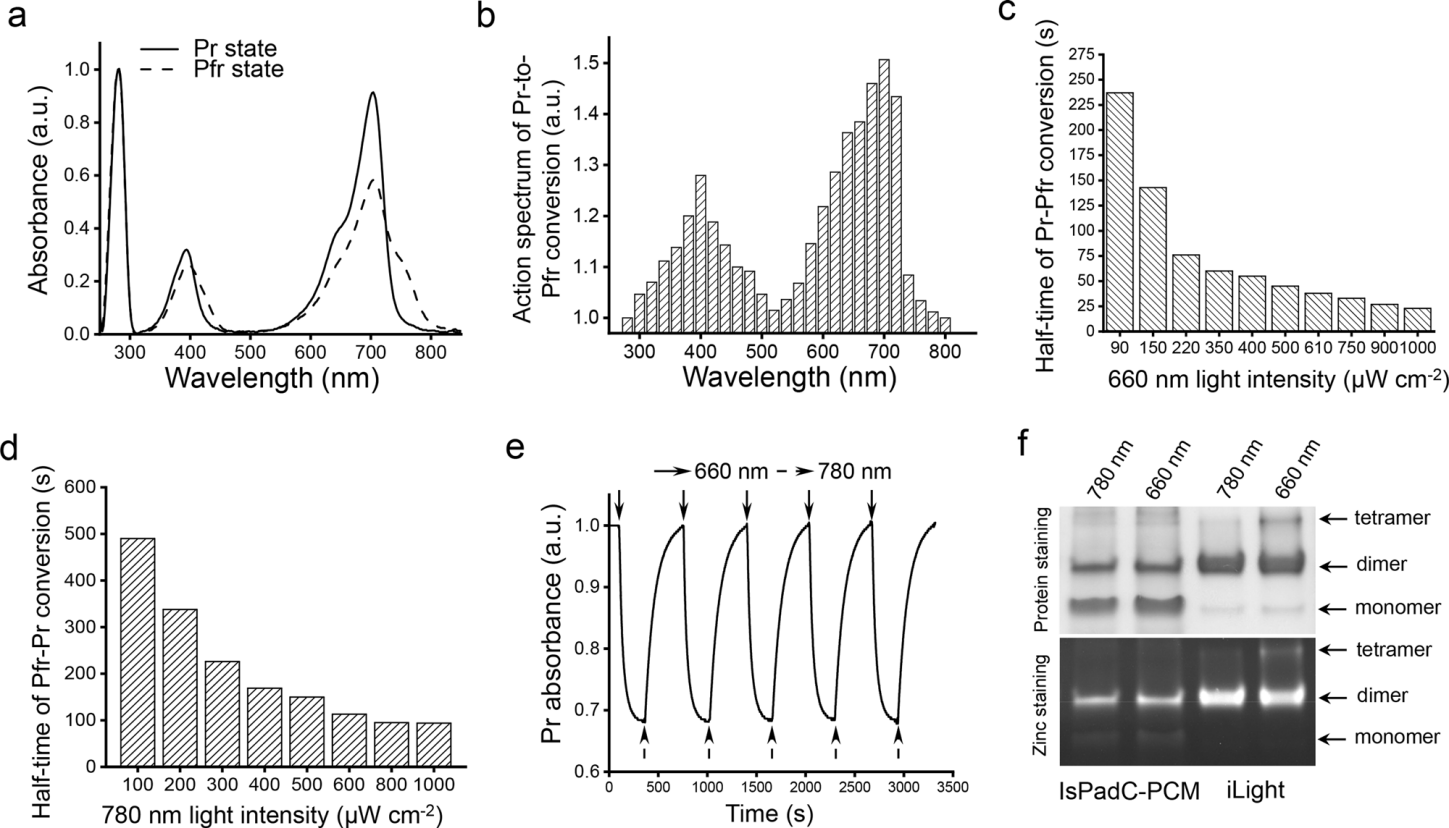
Spectral and photochemical properties of the purified iLight protein. **a** Absorbance spectra of iLight in the ground Pr state (solid line) and after the photoconversion to the Pfr state (dashed line). **b** Action spectrum of the Pr → Pfr photoconversion upon irradiation with light of specific wavelength measured as the relative decrease of the Pr state absorption at 704 nm. **c** Dependence of the half-time of the Pr → Pfr photoconversion on the intensity of 660/15 nm light. **d** Dependence of the half-time of the Pfr → Pr photoconversion on the intensity of 780/30 nm light. **e** Absorbance of iLight in the Pr state during repeated illumination cycles with 660/15 nm light and then with 780/30 nm light. Absorbance was measured at 704 nm. **f** Native PAGE of wild-type IsPadC-PCM and iLight. Top: proteins were illuminated with either 780 nm (photoconverting to the Pr state) or 660 nm light (photoconverting to the Pfr state) for 30 min and then run at 20 µg of the protein per lane in darkness. Bottom: ZnCl_2_ staining of the same gel visualizes the amount of the biliverdin chromophore covalently bound to each oligomeric fraction of the proteins. Arrows indicate the bands of the respective oligomeric states of the proteins. Experiment was independently repeated three times with similar results. In **a**, **b**, **e**, a.u., arbitrary units.

iLight originates from canonical IsPadC BphP that adopts Pr form as a ground state^19^. In its ground state, the iLight variant absorbed at 394 nm (Soret band) and 704 nm (Q band) (Fig. 3a). Upon 660 nm illumination, it photoconverted into the Pfr state with absorption maxima at 396 nm Soret band and notable shoulder at 752 nm corresponding to Q band. We also observed a 1.6-fold decrease in absorbance at 704 nm in the Pfr state, similar to wild-type IsPadC^19^. The Pr → Pfr photoconversion can be achieved by ~640–720 nm light, with the maximum photoconversion efficiency at 700 nm (Fig. 3b). The half-time of the Pr → Pfr photoconversion (half of the protein was converted) was 23 s at 1000 µW cm^−2^ and increased to 237 s at 90 µW cm^−2^ (Fig. 3c).

iLight returns from the Pfr state back to the ground Pr state after dark relaxation or after illumination with 780 nm light. The kinetics of Pfr → Pr dark relaxation was substantially slower than with 780 nm light (Supplementary Fig. 8). Moreover, the iLight dark relaxation was substantially slower than that of wild-type IsPadC-PCM (Supplementary Fig. 9). The half-time of the Pfr → Pr photoconversion of iLight depended on the 780 nm light intensity, ranging from 91 s at 1000 µW cm^−2^ to 490 s at 100 µW cm^−2^ (Fig. 3d). Multiple cycles of the reversible Pr-Pfr photoswitching did not lead to notable changes in the iLight absorbance at 704 nm (Fig. 3e).

Native PAGE followed by Zn^2+^ staining for biliverdin chromophore (Fig. 3f and Supplementary Fig. 10 with full gel) and size-exclusion chromatography (Supplementary Fig. 11) indicated that in the Pr state iLight exists as a dimer. Its photoconversion to the Pfr state with 660 nm light causes the formation of a tetrameric fraction. In contrast, wild-type IsPadC-PCM did not form a notable tetrameric fraction after 660 nm illumination. These data suggest a dimer-to-tetramer formation as the mechanism of action of the iLight-based optogenetic constructs.

**Fig. 4:**
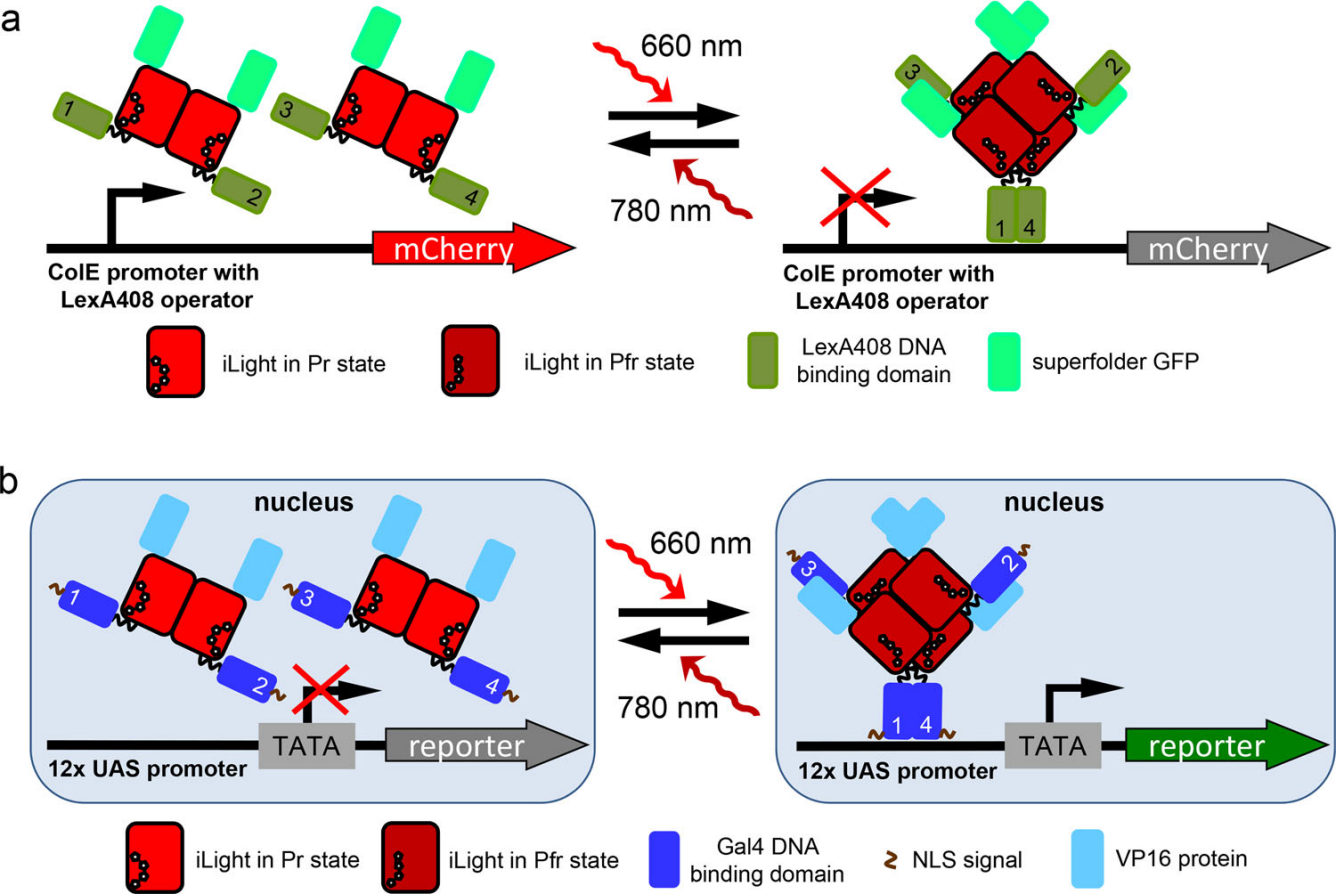
Proposed mechanism of action of the iLight optogenetic systems. **a** Schematics of the mCherry gene transcription repression in the iLight bacterial system. Expression of mCherry from the constitutively active ColE promoter with LexA408 operator is controlled by the oligomeric state of iLight fused with DNA-binding domain of LexA408. **b** Schematics of the reporter gene transcription activation in the iLight mammalian system. To induce reporter expression from the plasmid with 12× (upstream activating sequence) UAS upstream of minimal promoter, a nucleus localization signal (NLS)-tagged iLight was fused with a Gal4 DNA-binding domain and a VP16 transcriptional activation domain. The 660 nm light-induced iLight tetramerization brings the Gal4 DNA-binding domains into proximity, enabling them to bind 12× UAS and allowing VP16 to recruit transcription initiation complexes.

It has been shown that similar to other canonical BphPs, wild-type IsPadC and IsPadC-PCM form the tight parallel dimers^19,23^. However, unlike other BphPs in which N-termini extended from the PAS domain are typically unstructured, an N terminus of IsPadC in the Pr ground state forms an α-helical structure. Moreover, it is turned by its N terminus towards the PHY domain because of the interaction with the tongue structure of the PHY domain^23^. In the Pr state, the PHY-tongue forms two anti-parallel β-sheets^24^. Moreover, unlike other BphPs, 660 nm light causes complete Pr → Pfr transition and the PHY-tongue restructuring into an α-helix in only one protomer of the IsPadC-PCM dimer^19,23^. Another IsPadC-PCM protomer in the photoactivated dimer still remains in the Pr state. However, the α-helix of the PHY-tongue in the photoactivated Pfr-state protomer is unable to interact and, consequently, stabilize the N-terminal helical structure, causing its partial unfolding and turning by almost 180° away from the PHY-tongue^19,23^. Likely, these structural features of IsPadC-PCM do not allow to bring close two DBDs fused to the N terminus of each protomer in the photoactivated IsPadC-PCM dimer. To achieve that, two dimers should be assembled and then photoactivated, providing a possibility for two DBDs, one from each dimer, to form an active transcription factor dimer at the DNA sequence. Apparently, this proposed mechanism of action is implemented in the iLight optogenetic system (Fig. 4a).

### iLight-induced transcription activation in mammalian cells

It has been shown that a GAL4-VP16 fusion protein efficiently activates gene transcription in mammalian cells by binding to repeats of yeast-derived upstream activation sequence (UAS)^25^. The GAL4-UAS gene transcription system is widely used in model organisms including insects, fishes, and mammals^26^.

**Fig. 5:**
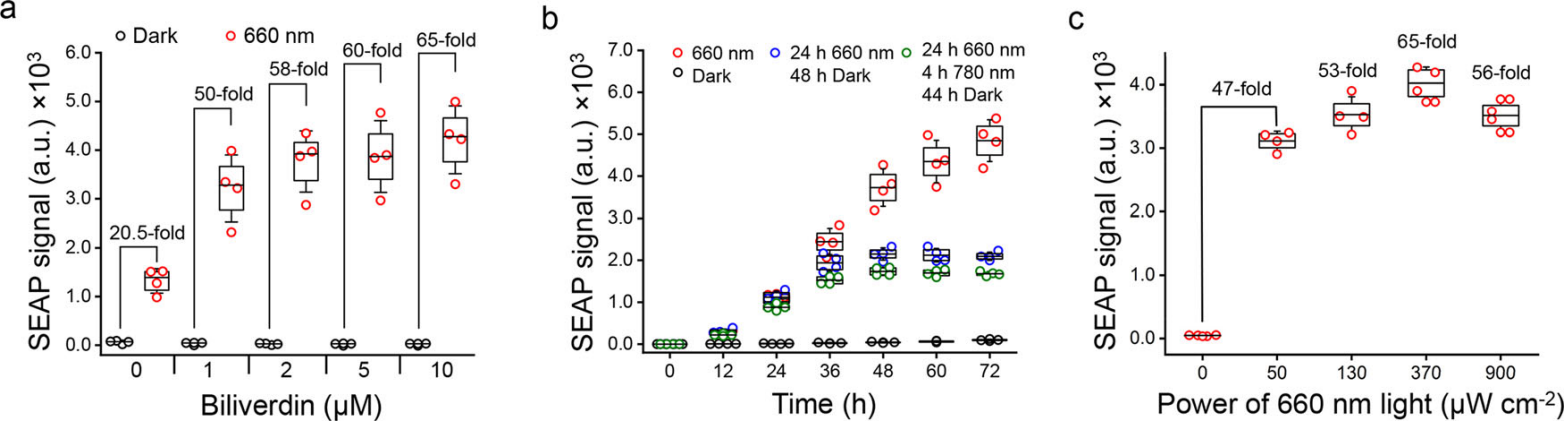
iLight-induced gene transcription activation in mammalian cells. **a** Dependence of the SEAP reporter expression on concentration of the exogenous biliverdin in HeLa cells expressing the iLight optogenetic system. SEAP signal was detected after 48 h of 660 nm illumination. **b** The 72 h-long kinetics of the SEAP reporter expression in HeLa cells with the iLight optogenetic system. Cells were illuminated in one of the following regimes: cells were kept in darkness (black dots); illumination with 660 nm light for 72 h (red dots); illumination with 660 nm light for 24 h followed by 48 h of darkness (blue dots); or illumination with 660 nm light for 24 h followed by 4 h of 780 nm light and 44 h of darkness (green dots). The culture medium samples were collected every 12 h to measure the SEAP signal. **c** Dependence of the SEAP reporter expression on the power of 660 nm activation light. HeLa cells were kept in darkness or under 660 nm light of the respective light intensities. Numbers indicate fold increase in the SEAP signal over darkness. In **b**, **c**, cells were supplemented with 10 µM biliverdin. In **a**–**c**, HeLa cells bearing NLS-Gal4-DBD-iLight-VP16 were transfected with pG12-SEAP (12× UAS) reporter plasmid. Box plots show the median (center line), first and third quartiles (box edges), 1× the SD (whiskers), and individual data points. *n* = 4 independent experiments for all conditions. a.u., arbitrary units. Source Data are available as a Source Data file.

To develop a light-inducible gene transcription system with iLight in mammalian cells, we fused a DBD (N-terminal 1–65 amino acid residues) of the yeast activator GAL4 (Gal4-DBD) to the N terminus of codon-optimized iLight and VP16 was fused to the C terminus of iLight (Fig. 4b and Supplementary Fig. 12). HeLa cells stably expressing NLS-Gal4-DBD-iLight-VP16 were transfected with pG12-SEAP reporter plasmid containing 12× UAS repeats upstream of secreted alkaline phosphatase (*SEAP*) gene. Illumination (660 nm) for 48 h increased the SEAP production by ~20.5-fold as compared to the cells in darkness without the supply of exogenous BV and by ~65- to 70-fold in cells supplemented with 10 µM of BV (Fig. 5a). The time course of SEAP production revealed ~19-fold SEAP increase after 24 h and up to ~70-fold after 72 h of 660 nm illumination as compared to the dark-treated cells (Fig. 5b).

We next studied how fast the light-induced transcriptional activation could be terminated. We illuminated cells with 660 nm light for 24 h and then kept them in darkness. The SEAP reporter production increased ~1.7-fold during the first 12 h in darkness, likely due to pre-accumulation of SEAP’s mRNA, and then the SEAP level stabilized (Fig. 5b). Similar SEAP kinetics was observed for cells illuminated with 780 nm light for 4 h right after the 24 h illumination period with 660 nm light. We observed ~1.7-fold increase of SEAP reporter production during the first 12 h after 660 nm illumination, without any following increase (Fig. 5b).

We further tested the dependence of the SEAP expression on the 660 nm light intensity (Fig. 5c). After an initial increase of the SEAP production in the 0–50 µW cm^−2^ light intensity range, the SEAP level reached a plateau in the 50–900 µW cm^−2^ range.

### Characterization of iLight optogenetic system in primary neurons

To characterize the system in neurons, we constructed an AAV vector expressing iLight system and the reporter AAV vectors expressing mCherry fluorescent protein and CheRiff channelrhodopsin^27^. In all vectors, the gene expression was driven by the calcium/calmodulin-dependent kinase II (CaMKII) promoter commonly used to express proteins specifically in cortical and hippocampal excitatory neurons. The neurons were isolated from hippocampi of newborn mice, cultured on glass coverslips, and transduced on a day in vitro 7 (DIV7) with iLight system and reporter AAVs. After the co-transduction, the neurons were kept in darkness with 2 μM of BV. On DIV12, the cells were illuminated with 660 nm light (500 µW cm^−2^, 30 s On, 180 s Off) to induce reporter expression. The illumination continued for 5 days and the cells were imaged afterward.

**Fig. 6:**
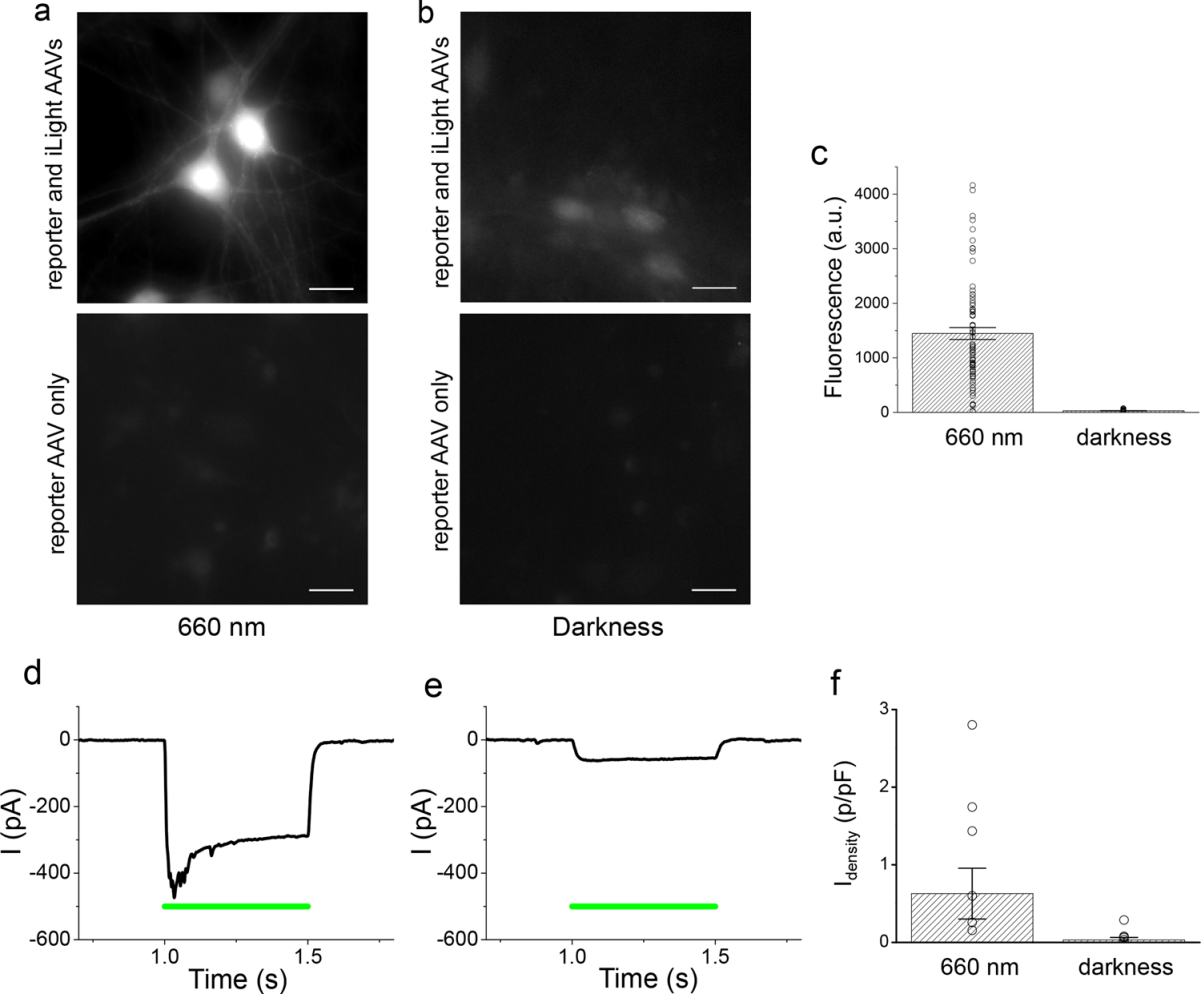
iLight-induced gene transcription activation in primary cultured neurons. Murine hippocampal neurons were co-transduced with the iLight optogenetic system and the mCherry and CheRiff reporter AAVs. **a**, **b** Representative images of the neurons transduced with iLight system and mCherry AAVs (top images) or with mCherry reporter AAV only (bottom images) incubated for 5 days either under 660 nm light (**a**) or in darkness (**b**). Scale bar, 20 μm. Experiments (**a**, **b**) were independently repeated three times with similar results. **c** Averaged mCherry reporter fluorescence in neurons cultured under 660 nm light (*n* = 53 cells) or in darkness (*n* = 62 cells), after subtraction of average fluorescence in cells transduced with mCherry AAV alone. The difference between groups was significant (paired two-sided Student’s *t*-test, exact *P*-values: *T* = 6, df = 113, *P* = 2.3 × 10^−8^). The data from typical experiment are presented. Error bars, SEM; a.u., arbitrary units. **d**, **e** Representative photocurrent traces in neurons co-transduced with iLight system and CheRiff reporter AAVs incubated either under 660 nm light (**d**) or in darkness (**e**) and exposed to 0.5 s of 505 nm light (green line) of 200 mW cm^−2^ during recording. **e** The cell responds with small photocurrent, because iLight system is inactive in darkness. Neurons in **d**, **e** were voltage-clamped at −70 mV and the photocurrent traces were smoothed by moving average filter with 2 ms window. **f** Effect of iLight system on photocurrent densities (current normalized by membrane capacitance) in the neurons expressing CheRiff and incubated either under 660 nm light or in darkness (*n* = 10 cells in each group). The difference between the two groups of neurons was significant (paired two-sided Student’s *t*-test, exact *P*-values: *T* = 2.17, df + 18, *P* = 0.044). Average photocurrents in the cells expressing CheRiff alone, without iLight system, were subtracted before statistical analysis. Error bars, SEM. Culture medium contained 2 μM biliverdin. Source Data are available as a Source Data file.

Bright fluorescence of the mCherry reporter was observed in neurons illuminated with 660 nm light (Fig. 6a), whereas in neurons kept in darkness the fluorescence intensity was substantially lower (Fig. 6b). The mCherry fluorescence levels varied substantially between individual cells exposed to 660 nm light (Supplementary Fig. 13). Similar high variability was observed in experiments in which neurons were transduced with AAV encoding another fluorescent protein driven by constitutive CaMKII promoter (Supplementary Fig. 14). After subtraction of the fluorescence levels in the control neuronal cultures transduced with mCherry reporter alone, the light-induced mCherry expression was significantly higher than in darkness (1446 ± 956.7 arbitrary units under light, 28.9 ± 23.9 arbitrary units in darkness, *T* = 6, df = 113, *p* = 2.3 × 10^−8^) (Fig. 6c). The results showed that the iLight optogenetic system can induce protein expression in primary cultured cells, such as hippocampal neurons.

### Multiplexing of iLight optogenetic system with channelrhodopsin

The absorption spectrum of iLight does not overlap with the activation spectrum of CheRiff channelrhodopsin, which is peaked at ~460 nm (Supplementary Fig. 15). Consequently, in neurons co-transduced with the iLight system and CheRiff reporter AAVs, the 660 nm illumination did not cause photocurrents and did not depolarize the cells (Supplementary Fig. 16). We tested whether the expression of CheRiff in neurons can be controlled with iLight system in the same way as the expression of mCherry. The neurons co-transduced with iLight and CheRiff were illuminated with 660 nm light (500 µW cm^−2^) starting on DIV12 (control culture was kept in darkness) and photocurrents induced by 505 nm light were measured on DIV17.

As expected, all patch-clamped neurons fired action potentials when the current (150–300 pA) was injected through the patch electrode (Supplementary Fig. 17). For photocurrent measurements, the neurons were held at −70 mV in voltage clamp mode. The 1 s flashes of 505 nm light of 200 mW cm^−2^ activated CheRiff and induced photocurrents in all transduced neurons (example cell current traces are shown in Fig. 6d, e). The resulting steady-state photocurrent values were divided by the values of cell membrane capacitance to obtain a current density, which is an estimate of the number of functional channelrhodopsin molecules per unit of the cell surface. The photocurrent density reached 2–3 pA/pF in some cells. Average photocurrent density in neurons kept under light was significantly higher than in cells kept in darkness (0.82 ± 0.33 and 0.11 ± 0.03 pA/pF, respectively, 10 cells in each group, *T* = 2.17, df = 18, *p* = 0.044). After subtraction of average photocurrent density values in cells transduced with CheRiff alone (0.19 ± 0.06 under light, 0.08 ± 0.03 pA/pF in darkness, 5 cells in each group), the average photocurrent density increase due to iLight-mediated activation was 20.1-fold higher in neurons incubated under 660 nm light than in the cells kept in darkness (Fig. 6f). These experiments validated that the iLight optogenetic system can drive expression of channelrhodopsin actuator in neurons, enabling crosstalk-free spectral multiplexing with optogenetic tools activated by blue–green light.

### iLight-driven light-activation of protein expression in vivo

**Fig. 7:**
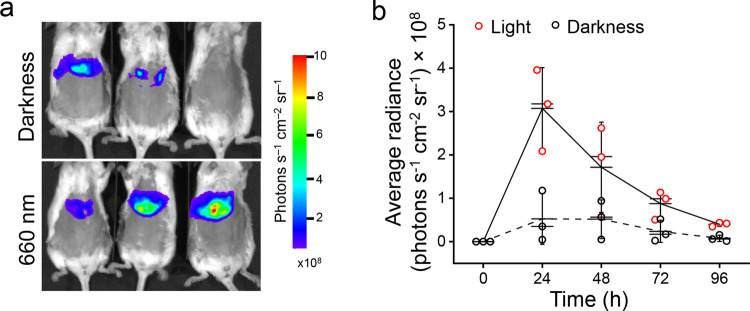
iLight-induced gene transcription activation in mouse tissue in vivo. **a** RLuc8 luciferase reporter signals detected in mice after the hydrodynamic co-transfection of the livers with the NLS-Gal4-DBD-iLight-VP16 and pG12-RLuc8 plasmids. Mice kept in darkness (top) or illuminated with 660 nm light of 3.2 mW cm^−2^ (bottom) for 48 h are shown. **b** Kinetics of the RLuc8 reporter expression in mice shown in **a** kept in darkness or illuminated for up to 96 h. Box plots show the median (center line), first and third quartiles (box edges), 1× the SD (whiskers) and individual data points. *n* = 3 individual animals. Source Data are available as a Source Data file.

For activation of gene transcription activation in deep tissues, we assessed the kinetics of light-induced *Renilla reniformis* luciferase (RLuc8) expression in the livers of mice, which were hydrodynamically co-transfected with the plasmid encoding NLS-Gal4-DBD-iLight-VP16 construct and the pG12-RLuc8 reporter plasmid (Fig. 7a). The maximum of the RLuc8 signal in the livers was observed after 24 h of 660 nm illumination. The RLuc8 signal was ~6-fold higher in the illuminated mice than in the mice kept in darkness (Fig. 7b). The difference of the RLuc8 expression between the illuminated and dark-treated animals was observed up to 96 h after the hydrodynamic transfection. These results showed that the iLight optogenetic system performed well in mouse tissues in vivo and achieved the maximum contrast twice faster (24 h) than the heterodimerization-based RpBphP1–RpPpsR2 two-component system (48 h)^16^.

## Discussion

To engineer the light-controlled iLight variant of IsPadC-PCM, we have developed the directed molecular evolution approach in which light-induced change of oligomeric state of the IsPadC-PCM mutants resulted in the dimerization of LexA408-DBD domains and consequent repression of the reporter protein production. Notably, in this approach, the DNA-binding domain of LexA408-mutated repressor and its operator are orthologous in *E. coli* cells and do not affect endogenous processes.

Structural^19,23^ and biochemical (Fig. 3f and Supplementary Fig. 11) analyses favor the tetramerization mechanism of action of the iLight system, with the I68F, R259H, and L464V amino acid substitutions being the most important for the system performance (Fig. 2e). Based on the crystal structure of the IsPadC (PDB ID: 6ET7)^23^, the Ile68 residue is located in the long, bent, uninterrupted α-helix of the PAS domain (Supplementary Fig. 18). According to the HDX-MS analysis^19^, the PAS domain of IsPadC does not directly contribute to the Pr-Pfr transition, suggesting that I68F substitution adds to the overall iLight rigidity and may facilitate the BV incorporation. The Arg295 residue is located at the GAF-proximal terminal part of the GAF-PHY α-helix and positioned in proximity to the α-helix of the GAF domain of another protomer in the IsPadC dimer. Possibly, R295H mutation may be involved in the light-induced interaction of the two iLight dimers, resulting in their tetramerization.

The Leu464 residue is located in the conserved in BphPs ^464^LXPRXSF^470^ amino acid motif of the PHY-tongue^24^, which is involved in the stabilization of the Pr and Pfr states^28^. Likely, in IsPadC Leu464 accelerates the docking of Arg467 with Asp207 and Tyr263 surrounding the biliverdin chromophore during the Pfr → Pr transition^29^. Interestingly, an Agp1 BphP from *Agrobacterium tumefaciens* contains Ile in the same position and has slow dark reversion to the ground Pr state^30^. Similarly, iLight exhibits a significantly reduced relaxation rate in darkness as compared to wild-type IsPadC-PCM (Supplementary Fig. 9). Apparently, iLight benefits from the slow Pfr → Pr dark reversion, because the dissociation of the iLight tetramers is delayed, which results in longer association of the DBDs and, consequently, their interaction with the corresponding DNA sequence. Not surprising that the mutation at position 464 has appeared early in the molecular evolution of iLight (Supplementary Table 1).

We hypothesized that the other six amino acid substitutions observed in iLight (Supplementary Table 1) improved the protein folding and the BV binding, which is needed for the formation of the IsPadC-PCM dimer (Fig. 3f).

We successfully applied the mammalian iLight optogenetic system to light-activate expression of the reporter proteins under human cytomegalovirus (CMV) promoter in conventional HeLa cells and under neuron-specific CaMKII promoter in primary neurons. Experiments in HeLa cells provided up to 65- to 70-fold increase in the production of the SEAP reporter but had limited reversibility (Fig. 5b). In contrast, the multiple cycles of photoswitching between the Pr and Pfr states were observed with the purified iLight protein (Fig. 3). Likely, the apparent irreversibility in the mammalian cells was caused by several factors, including high stability of the SEAP’s mRNA and high-affinity interaction of the dimerized via tetrameric iLight Gal4 DBDs with the UAS repeats. To clarify this issue, in future studies one may need to vary the stability of the mRNA encoding reporter and to add degradation peptide sequence to the reporter. Moreover, further crystallization and structural analysis of iLight in the Pr and Pfr states should provide more details on the tetramerization mechanism underlining the functioning of the iLight optogenetic constructs.

In neurons, as CheRiff channelrhodopsin is activated by blue–green light (peak of activation at ~475 nm), 660 nm illumination used to induce iLight-mediated gene transcription did not affect CheRiff activity (Supplementary Fig. 16). The large spectral separation allowed combining channelrhodopsin actuator and iLight system in the same cells, resulting in efficient spectral multiplexing. As the CheRiff is not activated by a red light while being produced, this combination preserves natural neuronal activity until a sufficient amount of CheRiff molecules is expressed. This approach is more suitable than combining shorter wavelength light (e.g., 470 nm) for activation of gene expression^31^ and red light for activation of red-shifted channelrhodopsins, because channelrhodopsins sensitive to red light, such as VChR1, still retain responsivity to shorter wavelengths^32^. More generally, the spectral multiplexing enabled by the iLight system could be used for co-expression of other molecular tools activated or excited by blue–green light, including LOV- and CRY2-based optogenetic tools^33,34^ and biosensors based on GFP-derived fluorescent proteins^35^.

The photocurrents generated by CheRiff with short flashes of 505 nm light were sufficient to depolarize neurons and drive action potentials. The magnitude of the resulting current densities was comparable to that observed in CheRiff-expressing neurons in other studies (e.g., 2.8–4.4 pA/pF in the culture of dorsal root ganglion cells)^36^. Channelrhodopsins^32^ including CheRiff^36^ are widely used to stimulate spiking in neurons in the brains of various animals. Our results suggest that the iLight optogenetic system can be further applied to control neuronal activity in vivo. We hypothesize that the light-induced increase of the CheRiff-medicated photocurrent in neurons in the mouse brain could substantially enhance their firing.

The observed substantial increase of the RLuc8 reporter expression in the liver of mice (Fig. 7) indicates that the iLight optogenetic system can be used in deep tissue applications in vivo. NIR light that triggers iLight exhibits deeper tissue penetration, lower scattering, and lower phototoxicity than light in the visible range.

Until now, NIR optogenetic systems required multiple components to operate. One type of these systems is based on the heterodimerization of phytochrome and binding partner, such as plant phytochrome PhyB and PIF61 factor^10,37,38^, or bacterial phytochrome RpBphP1 and its binding partners RpPpsR2^16^ or QPAS1^18^. The other type is based on the engineered cascade with the bacterial phytochrome diguanylate cyclase BphS^39^ as a light-sensing module. This multi-component cascade is initiated by a second messenger c-di-GMP produced by BphS and requires the co-expression of c-di-GMP-sensitive transcription factor and c-di-GMP phosphodiesterase^40,41^. To apply both types of the optogenetic systems in vivo, three AAVs are required. In contrast, the iLight one-component system requires only two AAVs, as it was shown in neurons (Fig. 6), thus substantially simplifying its applications in vivo.

Probably, because of the smaller size of iLight (60 kDa) than two-component NIR systems (full-length dimeric phytochrome of 80 kDa and dimeric interacting partner of 50–60 kDa), the iLight-based construct is synthesized faster and, correspondingly, provides the maximal activation contrast (reporter expression level) twice faster (Fig. 7) than the two-component systems^16,17^.

Almost twice the larger activation contrast achieved by the iLight system in mammalian cells (65- to 70-fold) than that by the RpBphP1–RpPpsR2 and RpBphP1–QPAS1 systems under the similar conditions (35-40-fold)^16^ may result from the lower background activation in the darkness of iLight than RpBphP1. However, further time-resolved and structural studies of BV assembling time with the bathy BphP having the Pfr ground state are required to evaluate the influence of this transition period on the background level of the corresponding optogenetic systems in darkness.

## Methods

### Design of bacterial and mammalian plasmids

An *IsPadC* gene was kindly provided by A. Winkler (Graz University of Technology, Austria). A transcription activating domain of transactivating tegument protein VP16 from *Herpes simplex* and Gal4(1–65) DBD were PCR amplified from a pGV-2ER plasmid (Systasy). A *RLuc8* gene encoding modified luciferase from *R. reniformis* was PCR amplified from a Nano-lantern/pcDNA3 plasmid (Addgene #51970). A *SEAP* gene was PCR amplified from a pKM006 plasmid kindly provided by W. Weber (University of Freiburg, Germany).

Reporter plasmid for screening and selection of IsPadC-PCM mutants was based on pLEVI(408)-ColE plasmid^42^. The pLEVI(408)-ColE plasmid was kindly provided by Y. Yang (East China University of Science and Technology, China). Using quick-change mutagenesis nucleotide sequence encoding VVD was substituted with sites for SacII and SalI endonucleases. Next, SAGG-IsPadC-PCM (1–532 amino acids) was cloned using SacII and SalI restriction sites, and 2x(SGGG)-msfGFP was cloned using SalI and EcoRI restriction sites, resulting in plasmid encoding LexA408-DBD(1-87)-SAGG-IsPadC-PCM(1-532)-2x(SGGG)-msfGFP. pWA23h plasmid encoding heme oxygenase for biliverdin synthesis in *E. coli* was modified to provide an expression of heme oxygenase under control of the constitutively active promoter. The rhamnose-inducible promoter of pWA23h was substituted with a constitutively active β-lactamase promoter from the pUC19 plasmid, resulting in a pWA23h-bla plasmid.

The reporter plasmids pG12-SEAP and pG12-Rluc8 were obtained by cloning of the *SEAP* and *Rluc8* genes, respectively, by AgeI and NotI sites, into a pG12 plasmid synthesized by GeneScript. Plasmids encoding a PiggyBac transposase pRP[Exp]-mCherry-CAG>hyPBase (VectorBuilder #VB160216-10057) and transposon-bearing plasmid pQP-Select were kindly provided by T. Redchuk (University of Helsinki, Finland). These plasmids were used to develop a stable preclonal cell mixture of HeLa cells.

A modified pBAD/His-B (Life Technologies-Invitrogen) vector with a shorter linker between the N-terminal polyhistidine tag and the gene of interest was used for bacterial expression of the iLight protein.

Plasmid for expression of the optogenetic system in mammalian cells was based on the pEGFP-N1 vector with truncated CMVd1 promoter. Enhanced GFP (EGFP) was substituted with a nucleotide sequence encoding T2A-mTagBFP2 using XhoI and XmaI restriction sites. Nucleotide sequence encoding NLS-SGGGG-Gal4(1-65)-4x(SAGG)-iLight(human codon-optimized)-5x(SGGGG)-VP16 was synthesized by GenScript and cloned using NheI and XhoI restriction sites into pT2A-mTagBFP2-N1 vector.

To transduce neurons, plasmids were created for packaging of the nucleotide sequences encoding optogenetic system and reporter into AAV. The pAAV-CW3SL-EGFP plasmid (Addgene #61463) was used as a backbone. EGFP was replaced with nucleotide sequence encoding NLS-SGGGG-Gal4(1-65)-4x(SAGG)-iLight (human codon-optimized)-5x(SGGGG)-VP16 for optogenetic system. To make the reporter plasmid, the CaMKII promoter and EGFP were replaced with the nucleotide sequence of the minimal promoter with 12 UASs followed by the nucleotide sequence encoding CheRiff-T2A-mCherry.

The major plasmids designed in this study are summarized in Supplementary Table 2. The major oligonucleotide primers used in this study are summarized in Supplementary Table 3.

### Mutagenesis and directed molecular evolution

Random mutagenesis of IsPadC-PCM (1–532 amino acids) was performed with a GeneMorph II random mutagenesis kit (Stratagene) using conditions that resulted in the mutation frequency of up to 16 mutations per 10^3^ bp. After mutagenesis, a mixture of mutated genes was cloned into pLEVI(408)-ColE-msfGFP plasmid using SacII and SalI restriction sites, and electroporated into TOP10 host cells (Invitrogen) containing the pWA23h-bla plasmid facilitating BV synthesis. Typical mutant libraries consisted of more than 10^6^ independent clones. For flow cytometry enrichment of the libraries, the TOP10 cells were grown overnight at 37 °C in Luria-Bertani (LB) liquid medium supplemented with spectinomycin and kanamycin in darkness. Bacterial cells were washed with phosphate-buffered saline (PBS) and diluted with PBS to an optical density of 0.03 at 600 nm. The libraries were enriched with FACSAria (BD Biosciences, software v.8.0.1) fluorescence-activated cell sorter using 488 and 561 nm lasers for excitation, and 530/30 and 610/20 nm emission filters for the selection of msfGFP and mCherry double-positive cells. Supplementary Fig. 19 exemplifies the gating strategy of fluorescence-activated cell sorting (FACS) and analysis used in this study. The 5 × 10^5^ bacterial cells were rescued in Super Optimal broth with Catabolite repression (SOC) medium at 37 °C for 1 h and then grown in LB medium supplemented with spectinomycin and kanamycin in darkness. The cells after enrichment were grown in darkness to an optical density of 0.4 at 600 nm. After 200-fold dilution in LB medium supplemented with spectinomycin and kanamycin, cells were grown at 37 °C for 16 h under 660/15 nm light at 0.25 mW cm^−2^. Bacterial cells were washed with PBS and diluted with PBS to an optical density of 0.03 at 600 nm. The msfGFP-positive and mCherry-negative cells were collected using FACSAria fluorescence-activated cell sorter using 488 and 561 nm lasers for excitation, and 530/30 and 610/20 nm emission filters. The 1 × 10^5^ collected bacterial cells were rescued in SOC medium at 37 °C for 1 h and then grown on LB/spectinomycin/kanamycin Petri dishes at 37 °C in darkness. After 10 h of cultivation, each dish was replicated on two dishes using a replica-plating tool (Cole-Parmer). Then dishes were cultivated overnight at 37 °C in the darkness and under 660/20 nm light at 0.25 mW cm^−2^.

Screening for mutants on Petri dishes with a decreased level of mCherry expression under 660/15 nm illumination was performed with a Leica MZ16F fluorescence stereomicroscope equipped with 480/30 and 570/30 nm excitation filters, and 530/40 and 615/40 nm emission filters (Chroma). Images of two replica dishes grown in different conditions (darkness and under 660/20 nm illumination) were aligned using Template Matching and Slice Alignment ImageJ plugin^43^, and colonies with the highest ratio of darkness/illumination mCherry signal were selected for the next round of mutagenesis.

### Characterization of mCherry expression repression by IsPadC-PCM mutants in bacteria

Unless stated otherwise, all experiments were carried out in the *E. coli* strain TOP10 containing the pWA23h-bla plasmid facilitating biliverdin synthesis. The cells from frozen stock or bacterial streak bearing pLEVI(408)-ColE-IsPadC-PCM variant-msfGFP were grown at 37 °C in LB medium supplemented with spectinomycin and kanamycin under 660/20 nm light at 0.25 mW cm^−2^ until an optical density of 0.2–0.3 at 600 nm. Two milliliters of each bacterial culture diluted to an optical density of 0.002 at 600 nm were transferred to new 15 ml tubes and were cultivated at 37 °C in darkness or under illumination. After overnight cultivation, the mCherry fluorescence signal of bacteria grown in darkness or under illumination was measured using FACS or spectrofluorimeter. For FACS analysis, bacterial cells were washed with PBS and diluted with PBS to an optical density of 0.03 at 600 nm and analyzed using FACSAria (BD Biosciences) fluorescence-activated cell sorter equipped with 561 nm laser for excitation and 610/20 nm emission filter. Bacterial cells were washed with PBS and diluted with PBS to an optical density of 0.1 at 600 nm, to measure mCherry signal in bacterial suspension using excitation 530 nm, emission 560–750 nm with a FluoroMax-3 spectrofluorometer (Horiba/Jobin Yvon).

To perform time-course illumination and single-point mutation analysis, bacteria were incubated in LB liquid medium supplemented with spectinomycin and kanamycin in darkness overnight. The next day, cell suspension was transferred on LB plates with the same antibiotics. Plates were dried and immediately transferred to a 37 °C incubator either fully protected from light or illuminated with 660/15 nm LED (1 mW cm^−2^) using 30 s on and 3 min darkness cycles. After 24 h, plates were imaged with Leica MZ16F fluorescence stereomicroscope as described above. Immediately after imaging, the bacterial cells were resuspended in ice-cold PBS for flow cytometry analysis. Flow cytometry was performed using an LSRII flow analyzer (BD Biosciences) equipped with 488 and 561 nm lasers for excitation, and 530/40 and 610/20 nm emission filters, respectively. Typically 100,000 GFP-positive single cells were analyzed. To quantify cell fluorescence, a mean fluorescent intensity in the red channel was divided by the mean fluorescence intensity of the same population in the green channel.

### Photochemical and biochemical characterizations of iLight

For bacterial expression of the iLight, its nucleotide sequence was sub-cloned into a pBAD/His-D vector using KpnI and EcoRI restriction sites. Protein with 6× polyhistidine tags on the N terminus was expressed in BL21-AI bacteria (ThermoFisher Scientific, #C607003) containing the pWA23h plasmid for rhamnose-inducible BV synthesis. The bacteria were grown in LB medium supplemented with ampicillin, kanamycin and 0.02% rhamnose for 6–8 h at 37 °C followed by induction of the protein expression by adding of 0.1% arabinose and cultivation for 12 h at 37 °C and 24 h at 18 °C. Protein was purified using Ni-NTA agarose (Qiagen) according to the manufacturer’s protocol with minor modification. In elution buffer, 400 mM imidazole was substituted with 100 mM EDTA. After elution, the buffer was exchanged using a PD10 desalting column (GE Healthcare) or Amicon Ultra-15 centrifugal filter units (Millipore) if the additional concentration was required.

For absorbance measurements, a U-2000 spectrophotometer (Hitachi) was used. A photoconversion of the iLight variant containing proteins was performed with 660/15 nm and 780/30 nm custom-assembled LED sources in quartz microcuvette (Starna Cells). A determination of action spectrum was performed by measurement of changing in absorbance of Pr state of iLight variant at 704 nm upon illumination with photoconversion light. As a source of light, the FluoroMax-3 spectrofluorometer was used and the illumination time was normalized to the power of activation. A half-time of Pr → Pfr and Pfr → Pr transition was measured by registering absorbance at 704 nm, while illuminating with 660/15 and 780/30 nm light, respectively. All spectroscopic measurements were performed in PBS at room temperature.

For native PAGE, proteins were diluted to the concentration of 2 mg/mL in 20 mM HEPES pH 7.7, 300 mM NaCl buffer, and illuminated either with 660/15 or 780/30 nm light at 2 mW/cm^2^ intensity for 0.5 h. Twenty micrograms of protein samples were diluted in 2× loading buffer (125 mM Tris-HCl pH 6.8, 0.004% bromophenol blue, 2% glycerol) and immediately loaded on 4–20% gradient gel (BioRad). After 2 h of run in 1× Tris/Glycine running buffer without SDS, the gel was washed and incubated in 1 mM ZnCl_2_ solution for 1 h, imaged for zinc-dependent fluorescence excited with UV light, then stained with GelCode blue protein stain (BioRad).

Size-exclusion liquid chromatography of the Ni-NTA-purified proteins was performed in darkness using HiLoad 16/600 Superdex 200 column (GE Healthcare) at a flow rate of 1 ml/min. The column was equilibrated with 10 mM HEPES buffer pH 7.4 containing 150 mM NaCl, 10% glycerol, 50 μM EDTA, 1 mM dithiothreitol, 0.2 mM phenylmethylsulfonyl fluoride, 0.01% EP-40, and 0.2 mM benzodiazepine. The column was calibrated with BioRad gel filtration standards. The proteins were diluted to the concentration of 1.9 mg ml^−1^ in 20 mM HEPES pH 7.7, 300 mM NaCl buffer, and illuminated either with 660/15 or 780/30 nm light at 2 mW cm^−2^ intensity for 0.5 h before applying to the column.

### Mammalian cell culture

HeLa cells were grown in Dulbecco’s modified Eagle’s medium supplemented with 10% fetal bovine serum, penicillin–streptomycin mixture (all from Life Technologies-Invitrogen) at 37 °C in 5% CO_2_. Transient cell transfections were performed using an Effectene reagent (Qiagen).

Preclonal mixtures of HeLa cells were obtained using the plasmid-based PiggyBac transposon system. To this end, the desire for integration into genome sequences were cloned into the transposon-bearing plasmids pQP-Select and co-transfected with a plasmid encoding a hyperactive PiggyBac transposase. Cells were further selected with 700 μg ml^−1^ of G418 antibiotic for 2 weeks and enriched with FACSAria (BD Biosciences) fluorescence-activated cell sorter using 407 nm laser for excitation and 450/50 nm emission filter for selection of mTagBFP2-positive cells, resulting in the preclonal HeLa cell mixtures expressing NLS-Gal4(1-65)-iLight-VP16-T2A-mTagBFP2 under control of CMVd1 promoter.

To study transcription activation using iLight optogenetic system, HeLa cells stably expressing NLS-Gal4(1-65)-iLight-VP16-T2A-mTagBFP2 were transfected with a pG12-SEAP reporter plasmid and illuminated with 660/15 nm light.

### Secreted alkaline phosphatase assay

For SEAP detection in culture media, a Great EscAPe SEAP Fluorescence Assay kit (Clontech) was used. Twenty-five-microliter aliquots of cell culture media from wells of a 24-well plate were collected at each time point and stored at −20 °C. The fluorescence intensity of the SEAP reaction product was measured using the SpectraMax M2 plate reader (Molecular Devices).

### AAV production

High-titer AAV particles were obtained as described here^44^. Briefly, plasmid DNA for AAV production was purified with NucleoBond Xtra Maxi EF kit (Macherey-Nagel) and AAV-293 cells (Agilent Technologies) were co-transfected with AAV genome plasmid, pAAV-G12-mCherry-T2A-CheRiff (encoding reporters) or pAAV-CaMKII-GAL4-iLight-VP16 (encoding optogenetic system), AVV capsid plasmid pUCmini-iCAP-PHP.eB, and pHelper using polyethyleneimine (Santa Cruz). Cell media were collected 72 h after transfection. One hundred and twenty hours after transfection, cells and media were collected and combined with media collected at 72 h. Cells were collected by centrifugation and then lysed with salt-active nuclease (HL-SAN, Arcticzyme). Eight percent of polyethylene glycol (PEG) was added to media, incubated 2 h on ice, and then pelleted. PEG pellet was treated with SAN and combined with lysed cells. Cell suspension was clarified by centrifugation. The supernatant was applied on iodixanol gradient and subjected to ultracentrifugation 2 h and 25 min at 350,000 × *g*. Virus fraction was collected, washed, and enriched on Amicon-15 100,000 MWCO centrifuge device. Virus titer was defined by quantitative PCR (qPCR). An aliquot of the virus was consequently treated with DNAse I and proteinase K, and then used as a template for qPCR. A NheI-digested, pAAV-G12-mCherry-T2A-CheRiff, or pAAV-CaMKII-GAL4-iLight-VP16 plasmid with known concentration was used as a reference.

### Isolation and viral transduction of primary mouse neurons

Neurons were isolated from hippocampi of postnatal (P0–P1) Swiss–Webster mice using the protocol from ref. ^45^ and cultured in Neurobasal Plus Medium with B-27 Plus Supplement (Gibco), additional 1 mM GlutaMAX (Gibco), 100 U/ml penicillin, and 100 μg/ml streptomycin, on poly-d-lysine (EMD Millipore)-coated glass coverslips (thickness 0.13–0.17 mm, diameter 12 mm, ThermoFisher Scientific). Cell density was ~70,000 cells per coverslip. Half of the medium was exchanged twice a week. Neurons were transduced with AAVs on DIV7 (10^9^ viral genomes per well, medium volume 0.5 ml, in a 24-well plate). After transduction, 2 μM of BV was added.

### Characterization of optogenetic system in neurons

Neurons were transferred from darkness to 660 nm light (30 s On, 180 s Off cycle, 0.5 mW cm^−2^) on DIV12 (5 days after transduction) and recorded on DIV17 (10 days after transduction). Fluorescence of mCherry in neurons was measured using Olympus IX81 inverted microscope controlled by Micro-Manager 1.3 (Vale Lab, UCSF) and Matlab R2018b (MathWorks). The microscope was equipped with 585 nm LED (Mightex Systems), 650/45 nm excitation filter, 695LP dichroic mirror, 725/50 nm emission filter (Chroma), LUCPlanFLN 20×/0.45 NA objective (Olympus), Orca Flash 4.0LT camera, and HCImage software (Hamamatsu).

For the characterization of CheRiff expression, the steady-state ionic photocurrents were measured. We assumed that the channelrhodopsin expression level is proportional to the number of functional channelrhodopsin molecules per unit of cell membrane area and divided the photocurrent value (measured in pA) by the respective value of cell membrane capacitance (measured in pF and presumably proportional to cell membrane area). The neurons were patch-clamped in whole-cell configuration.

Patch pipettes were pulled from borosilicate glass with filament (OD 1.5 mm, ID 0.86 mm, Sutter Instruments) to resistance of 3–5 MΩ on P-1000 puller (Sutter Instruments). External bath solution contained 125 mM NaCl, 2.5 mM KCl, 1 mM MgC_2_, 10 mM HEPES, 3 mM CaCl2, 30 mM glucose pH 7.3, 305–307 mOsm. Internal solution contained 125 mM potassium gluconate, 8 mM NaCl, 0.6 mM MgCl_2_, 0.1 mM CalCl_2_, 1 mM EGTA, 4 mM MgATP, 0.4 mM NaGTP, 10 mM HEPES pH 7.3, 294–297 mOsm. Positive pressure (30–45 mbar) was maintained, while the pipette was approaching a cell. Gigaseal was established using 30–100 mbar negative pressure. For breaking the patch of the membrane, a pulse of −100 to −150 mbar negative pressure (duration ~50 ms) was applied concurrently with a single 1 V, 0.2 ms voltage pulse (“zap”). Voltage and current values were recorded and digitized with Intan CLAMP Patch Clamp Amplifier System at 50 kHz (Intan Technologies)^46^. Cell membrane capacitance was estimated by delivering square voltage pulses (10 mV, 50 ms duration, 50 Hz, holding voltage −70 mV), measuring resulting currents and fitting an exponential curve to the current trace. The estimation was performed automatically by Clamp UI software v.1.4.0 (Intan Technologies). Photocurrents were recorded in voltage clamp mode (−70 mV), while flashes of green light (duration 1 s, 505 nm LED, Mightex Systems, with 510/20 nm filter) were delivered. Values of resulting steady-state photocurrent were measured and divided by values of membrane capacitance to normalize photocurrents by cell membrane area. The timing of light pulses was controlled with Master-8 pulse stimulator (AMPI, Israel). Neuron images and traces of current and voltage were processed in Matlab R2018b (MathWorks).

### Hydrodynamic transfection of the liver in mice

The Swiss–Webster 2- to 3-month-old female mice (National Cancer Institute, NIH) with body weight of 22–25 g were used for delivery of plasmids encoding optogenetic system and reporter protein into the liver by hydrodynamic transfection. Ten micrograms of the pCMVd1-NLS-GAL4(1-65)-iLight-VP16-T2A-mTagBFP2 plasmid and 50 µg of the pG12-Rluc8 reporter plasmid in 1.5 ml of PBS were intravenously injected through a tail vein. The mice were placed in the cage without bedding and were illuminated from the bottom with the 660/20 nm LED array; control animals were kept in the darkness. Intensity of activation light was 3.2 mW cm^−2^. For better illumination and imaging, the belly fur was removed using a depilatory cream.

Animals were continuously illuminated or kept in darkness for 72 h, and every 12 h were released and fed for 30 min. Every 24 h after the hydrodynamic transfection, animals were imaged using an IVIS Spectrum instrument (Perkin Elmer/Caliper Life Sciences) in bioluminescence mode with an open emission filter. Throughout the imaging, animals were maintained under anesthesia with 1.5% vaporized isoflurane. Before imaging, 80 µg of Inject-A-Lume CTZ native (NanoLight Technology) were intravenously injected through a retro-orbital vein.

Data were analyzed using Living Image v.3.0 software (Perkin Elmer/Caliper Life Sciences). Specifically, the average signal from each animal was calculated from a region of interest located over the liver of the animal and each region of interest was of the same size.

All animal experiments were performed in an Association for Assessment and Accreditation of Laboratory Animal Care International-approved facility using protocols approved by the Albert Einstein College of Medicine Animal Usage Committee.

### Reporting summary

Further information on research design is available in the Nature Research Reporting Summary linked to this article.

## Supplementary information

Supplementary Information

Reporting Summary

## Data Availability

The data supporting the findings of this study are available within the article and its Supplementary Information. All other data that support the findings of the study are available from the corresponding author upon reasonable request. The major plasmids constructed in this study, their maps, and sequences are deposited to Addgene (#170268 - #170275). The iLight sequence is available at GenBank (MW890755). Source data are provided with this paper.

## Source data

Source Data File
