# Supplementary material for: Single-component near-infrared optogenetic systems for gene transcription regulation
Source: Nat Commun. 2021 Jun 23;12:3859. doi: 10.1038/s41467-021-24212-7 (PMC8222386; doi:10.1038/s41467-021-24212-7)
Supplement: Supplementary file 1 — Supplementary Information [file 41467_2021_24212_MOESM1_ESM.pdf]

Single-component near-infrared optogenetic systems for gene transcription regulation

Supplementary Information

Supplementary Table 1. Mutations in the IsPadC-PCM variants acquired during molecular evolution.

IsPadC-PCM variants	Amino acid mutations		
	**PAS domain**	**GAF domain**	**PHY domain**
**1.3**		E274K, R295H	I360V, L464M
**2.24**		T265I, E274K, R295H	I360V, L464M
**iLight**	I68F, H80Q, A86T, R90S	S242C, E274K, R295H	I360V, L464V

Supplementary Table 2. List of the major plasmids designed in this study.

Plasmid	Backbone	Promoter	Insert
pLEVI(408)-ColE-iLight-msfGFP	pLEVI(408)-ColE	J23116 promoter for iLight construct, and ColE with a LexA408 operator for mCherry reporter	iLight-msfGFP
pWA23h-bla	pWA23h	β-lactamase promoter	heme oxygenase
pBAD/His-D-iLight	modified pBAD/His-B	araBAD	iLight
pG12-SEAP	pG12	12xUAS with TATA-box minimal promoter	SEAP
pG12-Rluc8	pG12	12xUAS with TATA-box minimal promoter	RLuc8
pCMVd1-NLS-GAL4-iLight-VP16-T2A-mTagBFP2	pQP-Select	CMVd1	NLS-Gal4(1-65)-iLight(human codon optimized)-VP16-T2A-mTagBFP2
pAAV-CaMKII-GAL4-iLight-VP16	pAAV-CW3SL-EGFP	CaMKII	NLS-Gal4(1-65)-iLight(human codon optimized)-VP16

Supplementary Table 3. List of the major oligonucleotide primers used in this study.

Purpose	Primer	Sequence (5'-3')
Cloning of IsPadC-PCM or iLight into pBAD/His-D	forward	ttggtaccgcagcagatctgggtagtgatgat
	reverse	ttgaattcctaggtcagatcatcaaagctggccag
Cloning of IsPadC-PCM or iLight with linker into pLEVI(408)-ColE	forward	aaccgcggagtgctggtggggcagcagatctgggtagtgatgatatc
	reverse	aagaattcgctgtcgacggtcagatcatcaaagctggccag
Cloning of msfGFP with linker into pLEVI(408)-ColE	forward	gaccgtcgactccggtggtggttctggtggtggaatggtgagcaagggcgagg
	reverse	gaattctcacttgtacagctcgtccatgccgagagtgatccc
Cloning of SEAP into pG12	forward	cgtcaccggtcgccaccatgctgctgctgctgctgctgc
	reverse	catcgcggccgcttaacccgggtgcgcggcgtcg
Cloning of Rluc8 into pG12	forward	cgtaccggtcgccaccatggcttccaaggtgtacgaccccgagc
	reverse	ctggcggccgcttactgctcgttcttcagcactc
Cloning of iLight into mammalian vector.	forward	ttgaattcgccgccgacctgggctctgacgat
	reverse	gctggtaccgccgccagaggtcagatcgtcgaaggaggccag
Cloning of CheRiff with Kozak into pAAV-CW3SL-EGFP.	forward	ttggtaccggtcgccaccatgggcggagctcctgctc
	reverse	atctcgagccacgttgatgtcgatctggtccag
Introduction of F68I amino acid substitution into iLight.	forward	gataacaccattcatgaactgagcgatattaaacaggccaacattaatagcctgc
	reverse	gcaggctattaatgttggcctgtttaatatcgctcagttcatgaatggtgttatc
Introduction of Q80H amino acid substitution into iLight.	forward	cattaatagcctgctgccggaacatctgattagcggtctgacaagcgc
	reverse	gcgcttgtcagaccgctaatcagatgttccggcagcaggctattaatg
Introduction of T86A amino acid substitution into iLight.	forward	cggaacaactgattagcggtctggcaagcgcaattagtgaaaatgaacc
	reverse	ggttcattttcactaattgcgcttgccagaccgctaatcagttgttccg
Introduction of S90R amino acid substitution into iLight.	forward	gcggtctgacaagcgcaattcgtgaaaatgaaccgatttgggttg
	reverse	caacccaaatcggttcattttcacgaattgcgcttgtcagaccgc
Introduction of C242S amino acid substitution into iLight.	forward	caaaataccgaagcagttaatctgagcagcggtgttctgcgtgcagttag
	reverse	ctaactgcacgcagaacaccgctgctcagattaactgcttcggtattttg
Introduction of K274E amino acid substitution into iLight.	forward	cagcattggcatttttaacgaagatgaactgtggggtatcgttgcatg
	reverse	catgcaacgataccccacagttcatcttcgttaaaaatgccaatgctg
Introduction of H295R amino acid substitution into iLight.	forward	gcaattggtcgtcgtattcgtcgtctgctggttcgtaccgttgaatttg
	reverse	caaattcaacggtacgaaccagcagacgacgaatacgacgaccaattgc
Introduction of V360I amino acid substitution into iLight.	forward	ggtgtaaactgtttcgttgtgatggtattggttatctgcgtggagaagaac
	reverse	gttcttctccacgcagataaccaataccatcacaacgaaacagtttacacc
Introduction of V464L amino acid substitution into iLight.	forward	gaaaccagcactggcaccatgctgggtccgcgtaaaagttttg
	reverse	caaaacttttacgcggacccagcatggtgccagtgctggtttc

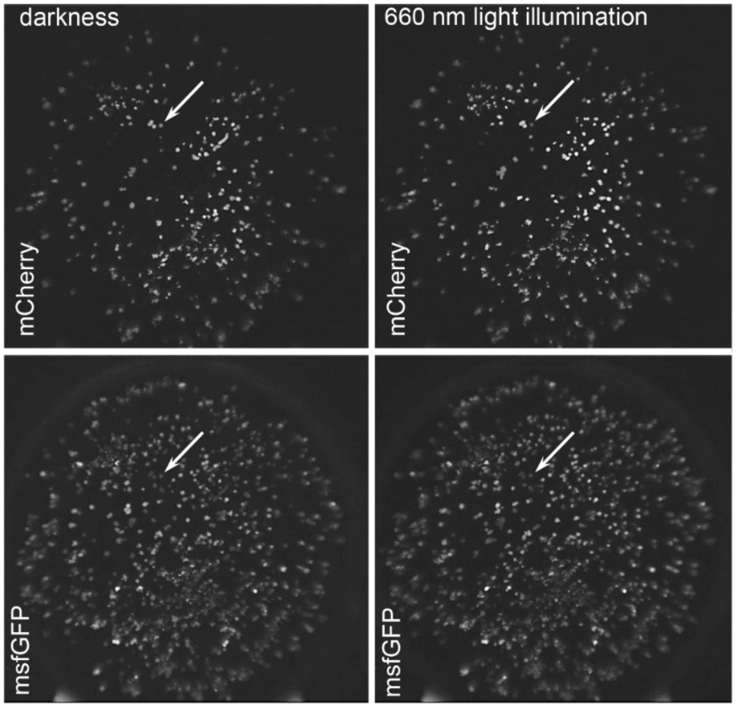

Supplementary Figure 1. Screening of FACS selected clones using replica approach. Replicated dishes were grown in the darkness or under 660 nm light. After overnight incubation dishes were imaged in mCherry and msfGFP and analyzed using ImageJ to find clones with maximum difference of signal in mCherry channel and minimum difference in msfGFP channel. White arrows indicates IsPadC-PCM clone 1.3 selected after 1-st round of mutagenesis with ~2-fold contrast of dark-to-light mCherry signal.

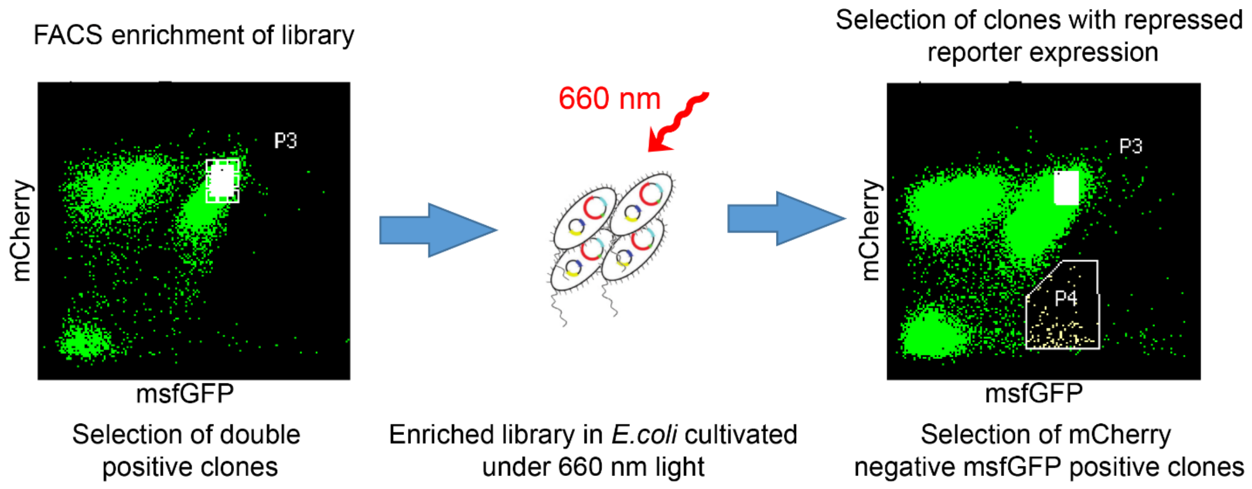

Supplementary Figure 2. Pipeline for selection of clones with light-activated repression of mCherry reporter expression. After random mutagenesis of IsPadC-PCM library of mutated variants were grown in E.coli bacteria in darkness with following enrichment of the double positive clones (gate P3 mCherry and msfGFP positive cells). Enriched library were overnight cultivated under 660 nm light and mCherry negative, msfGFP positive cells were selected (gate P4) for the following screening.

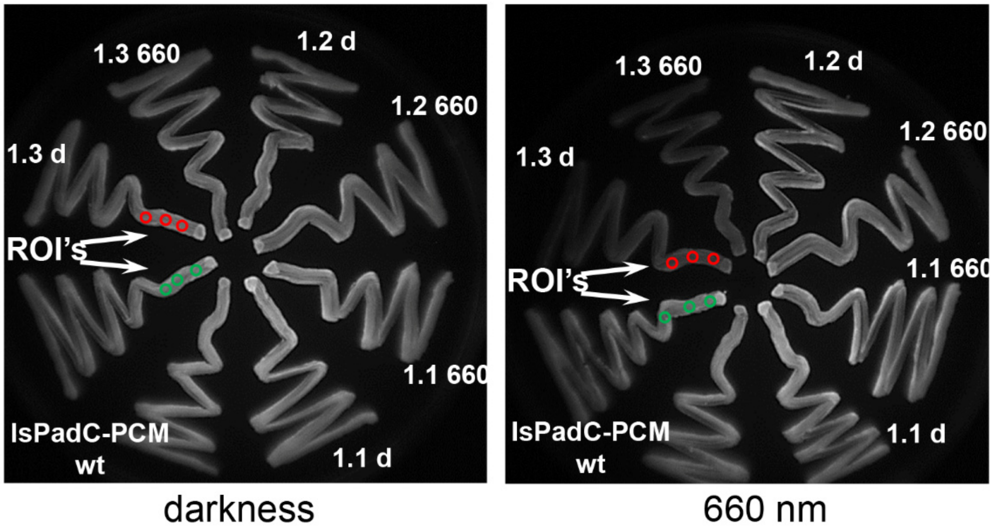

Supplementary Figure 3. Initial characterization of the selected IsPadC-PCM clones. Selected after screening clones (clones were selected from both dishes cultivated in the darkness and under 660 nm light) were streaked on dishes and cultivated overnight in the darkness or under 660 nm light. An intensity of mCherry signal was analyzed with ImageJ in the selected ROIs. The mCherry signal of the wild-type IsPadC-PCM streak was used as the reference.

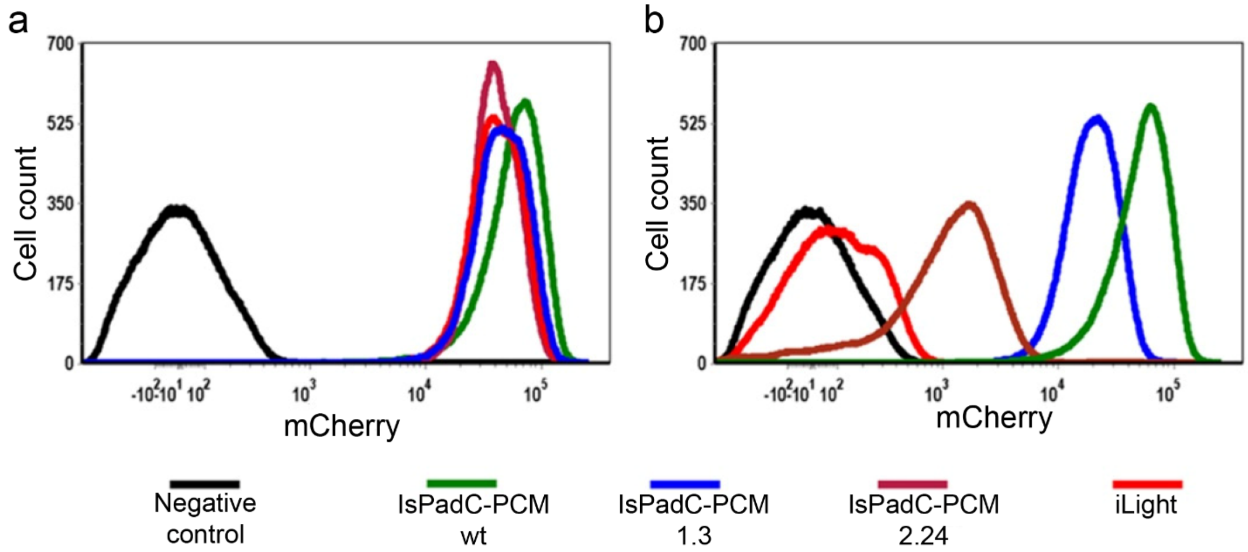

Supplementary Figure 4. Light-induced repression of mCherry expression in bacterial cells. FACS analysis of mCherry expression by TOP10 cells expressing IsPadC-PCM mutants selected after each round of mutagenesis cultivated in darkness (**a**) and under 660 nm light (**b**).

a LexA408:1-87--iLight--msfGFP

b MKALTARQQEVFDLIRDHISQTGMPPTRAEIAQRLGFRSASSAEEHLKALARKGVIEIVSGASRGIRLLQEEEEGLPLVGRVA
AGEPRSAGGAADLGSDDISKLIAACDQEPIHIPNAIQPFGAMLIVEKDTQQIVYASANSAEYFSVADNTIHELSDFKQANINS
LLPEQLISGLTSAISENEPIWVETDRLSFLGWRHENYYIIEVERYHVQTSNWFEIQFQRAFQKLRNCKTHNDLINTLTRLIQEI
SGYDRVMIYQFDPEWNGRVIAESVRQLFTSMLNHHFPASDIPAQARAMYSINPIRIIPDVNAEPQPLHMIHKPQNTEAVNLSC
GVLRAVSPLHMQYLRNFGVSASTSIGIFNEDKLWGIVACHHTKPRAIGRRIRHLLVRTVEFAAERLWLIHSRNVERYMVTVQ
AAREQLSTTADDKHQAHEIVIEHAADWCKLFRCDGVGYLRGEELTTYGETPDQTTINKLVEWLEENGKKSLFWHSHMLK
EDAPGLLPDGSRFAGLLAIPLKSDADLFSYLLLFRVAQNEVRTWAGKPEKLSVETSTGTMVGPRKSFEAWQDEVSGKSQPW
RTAQLYAARDIARDLLIVADSMQLNLLNDQLADANENLEKLASFDDLTVDSGGGSGGGMVSKGEELFTGVVPILVELDGD
VNGHKFSVRGEGEGDATNGKLTLKFICTTGKLPVPWPTLVTTLTYGVQCFSRYPDHMKRHDFFKSAMPEGYVQERTISFKD
DGTYKTRAEVKFEGDTLVNRIELKGIDFKEDGNILGHKLEYNFNSHNVYITADKQKNGIKANFKIRHNVEDGSVQLADHY
QQNTPIGDGPVLLPDNHYLSTQSKLSKDPNEKRDHMVLLEFVTAAGITLGMDELYK

Supplementary Figure 5. iLight optogenetic system for suppression of gene transcription in bacteria. (a) Components of the iLight fusion construct are highlighted with different colors. **(b)** Amino acid sequence of the corresponding parts of the iLight bacterial system shown in (a) is highlighted with the corresponding colors. Substitutions in iLight relative to wild-type IsPadC-PCM are indicated in red.

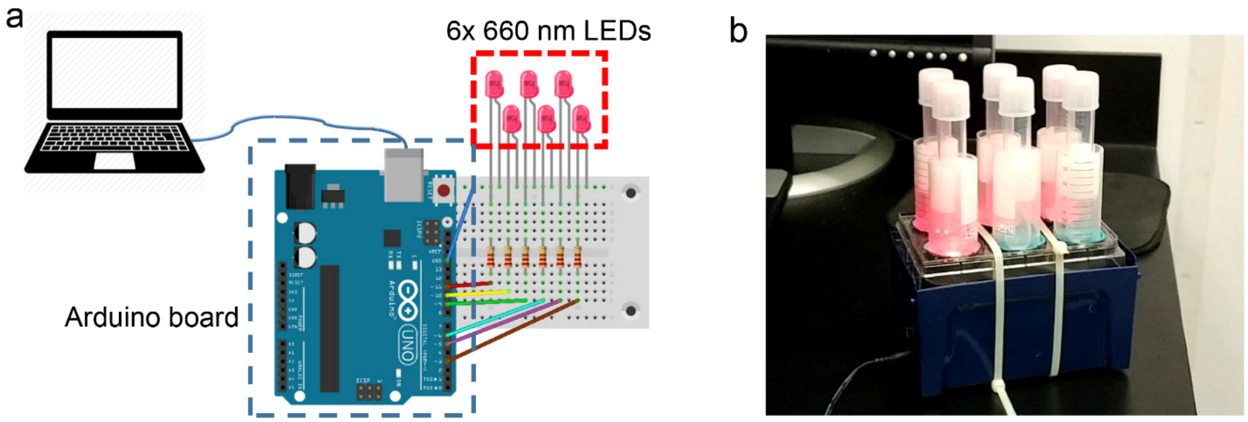

Supplementary Figure 6. Programmable multichannel 660 nm LED array. Principal scheme (**a**) and assambled prototype (**b**) of programmable 6-channel 660 nm LED array used for illumination of bacterial cells.

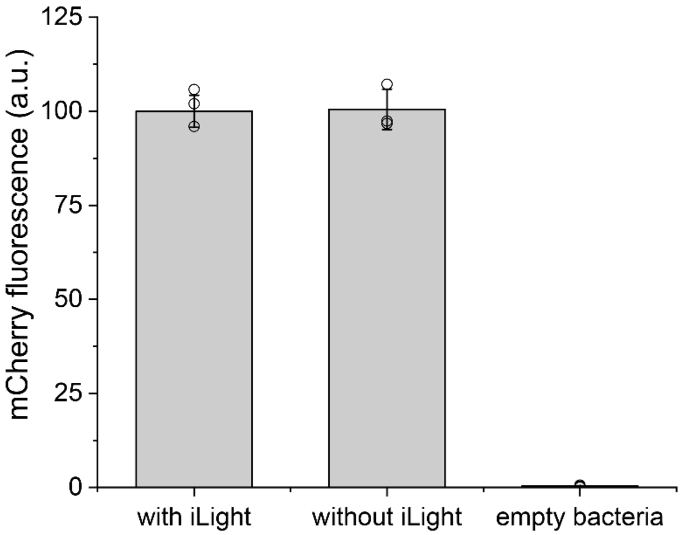

Supplementary Figure 7. Comparison of mCherry reporter levels in *E.coli* bacteria with or without iLight in darkness. The bacteria expressed either the full-length pLEVI(408)-ColE-iLight-msfGFP plasmid (left bar) or the same plasmid with the deleted iLight-msfGFP fragment (middle bar). The mCherry fluorescence intensity was the same in both types of bacteria, indicating that iLight did not decrease the expression level of the mCherry reporter. Control empty bacteria exhibited almost no fluorescence (right bar). Error bars, s.d. (n=3 independent experiments, a.u., arbitrary units).

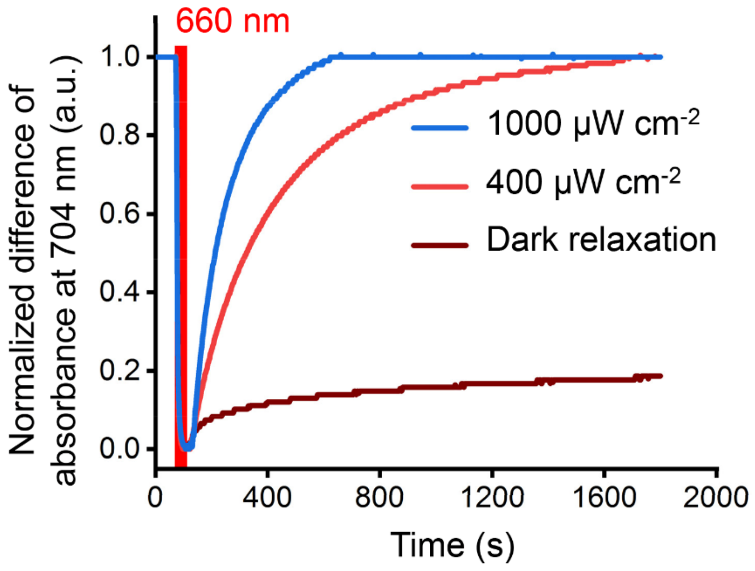

Supplementary Figure 8. Kinetics of iLight Pfr→Pr photoconversion *in vitro.* iLight was photoconverted into Pfr state with 660 nm light, and kinetics of Pfr→Pr photoconversion with 780 nm light or dark relaxation was measured by detecting of light absorbance at 704 nm. Absorbance is shown in arbitrary units, a.u.

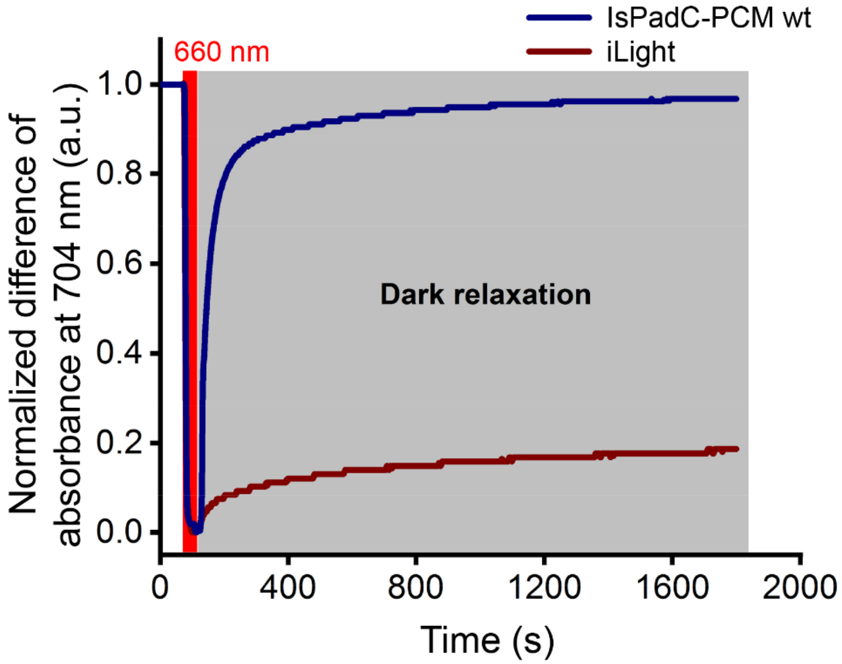

Supplementary Figure 9. Dark relaxation of wild-type IsPadC-PCM and iLight. Wild-type IsPadC-PCM and iLight were photoconverted to the Pfr state with 660 nm light, and dynamics of the dark relaxation were further monitored by detecting absorbance at 704 nm. The complete relaxation of iLight was observed after overnight incubation in darkness. Absorbance is shown in arbitrary units, a.u.

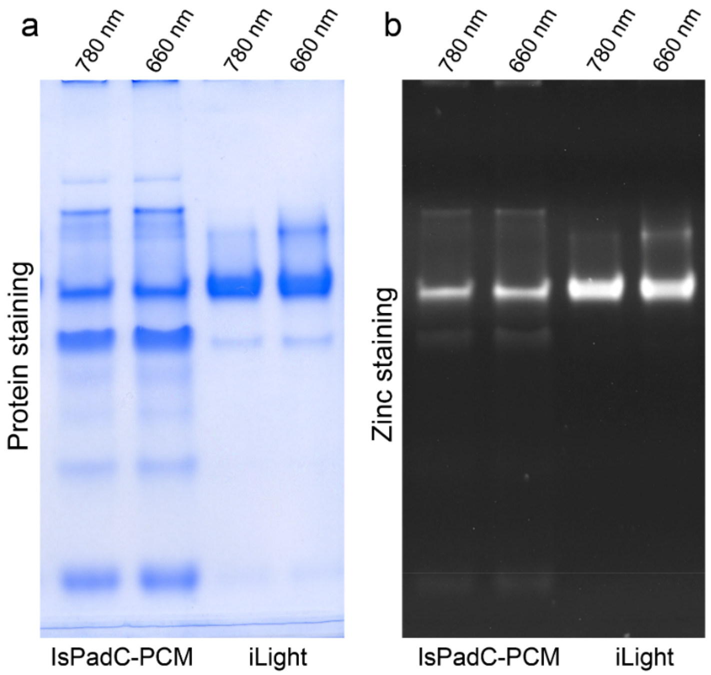

Supplementary Figure 10. Full native PAGE of IsPadC-PCM and iLight. (**a**) Proteins were illuminated with either 780 nm (photoconverting to the Pr state) or 660 nm light (photoconverting to the Pfr state) for 30 min and then run at 20 μg of the protein per lane in darkness. (**b**) $ZnCl_2$ staining of the same gel visualizes the amount of the biliverdin chromophore covalently bound to each oligomeric fraction of the proteins. See also Figure 3f. Experiments (a,b) were independently repeated 3 times with similar results.

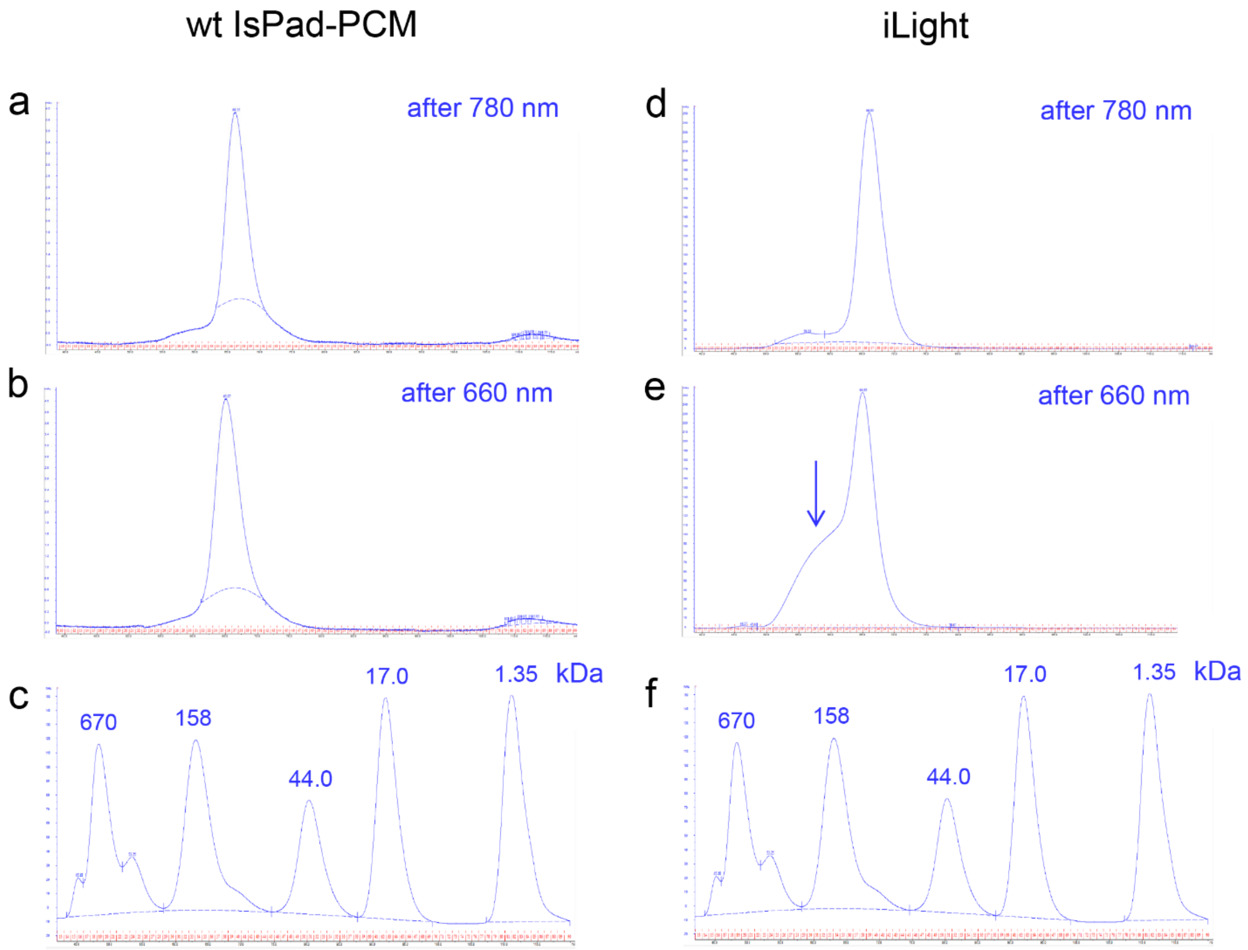

Supplementary Figure 11. Size-exclusion chromatography of the purified IsPadC-PCM and iLight proteins. (**a, b**) IsPadC-PCM and (**d, e**) iLight purified from bacteria were illuminated with either (a, d) 780 nm light (photoconverting it to the Pr state) or (b, e) 660 nm light (photoconverting it to the Pfr state) for 30 min at r.t. and then applied to size-exclusion chromatography at 1.9 mg/ml in darkness. The major elution peaks in (a, b, d, e) correspond to the protein dimers. After activation with 660 nm light ~25% of iLight protein elutes as a tetramer (arrow in (e)). (**c, f**) MW markers aligned with the elution profiles of IsPadC-PCM (c) and iLight (f).

a NLS--Gal4:1-65--iLight--VP16--T2A-- mTagBFP2

b MARKKKRKSGGGGKLLSSIEQACDICRLKKLKCSKEKPKCAKCLKNNWECRYSPKTKRSPLTRAHLTEVESRLERLEQTSAG
GSAGGSAGGSAGGEFAADLGSDDISKLIAACDQEPIHIPNAIQPFGAMLIVEKDTQQIVYASANSAEYFSVADNTIHELSDFKQA
NINSLLPEQLISGLTSAISENEPIWVETDRLSFLGWRHENYYIIEVERYHVQTSNWFEIQFQRAFQKLRNCKTHNDLINTLTRLIQ
EISGYDRVMIYQFDPEWNGRVIAESVRQLFTSMLNHHFPASDIPAQARAMYSINPIRIIPDVNAEPQPLHMIHKPQNTEAVNLSC
GVLRAVSPLHMQYLRNFGVSASTSIGIFNEDKLWGIVACHHTKPRAIGRRIRHLLVRTVEFAAERLWLIHSRNVERYMVTVQA
AREQLSTTADDKHQAHEIVIEHAADWCKLFRCDGVGYLRGEELTTYGETPDQTTINKLVEWLEENGKKSLFWHSHMLKEDA
PGLLPDGSRFAGLLAIPLKSDADLFSYLLLFRVAQNEVRTWAGKPEKLSVETSTGTMVGPRKSFEAWQDEVSGKSQPWRTAQL
YAARDIARDLLIVADSMQLNLLNDQLADANENLEKLASFDDLTSGGGTSGGGGSGGGGSGGGGSGGGGSGGGGSGGRSAYS
RARTKNNYGSTIEGLLDLPDDDAPEEAGLAAPRLSFLPAGHTRRLSTAPPTDVSLGDELHLDGEDVAMAHADALDDFDLDML
GDGDSPGPGFTPHDSAPYGALDMADFEFEQMFTDALGIDEYGGARDRRAEGRGSLLTCGDVEENPGPTSSELIKENMHMKLY
MEGTVDNHHFKCTSEGEGKPYEGTQTMRIKVVEGGPLPFAFDILATSFLYGSKTFINHTQGIPDFFKQSFPEGFTWERVTTYED
GGVLTATQDTSLQDGCLIYNVKIRGVNFTSNGPVMQKKTLGWEAFTETLYPADGGLEGRNDMALKLVGGSHLIANAKTTYRS
KKPAKNLKMPGVYYVDYRLERIKEANNETYVEQHEVAVARYCDLPSKLGHKLN

Supplementary Figure 12. iLight optogenetic system for gene transcription activation in mammalian cells. (a) Components of the iLight fusion construct are highlighted with different colors. **(b)** Amino acid sequence of the corresponding parts of the iLight mammalian system shown in (a) is highlighted with the corresponding colors. Substitutions in iLight relative to wild-type IsPadC-PCM are indicated in red.

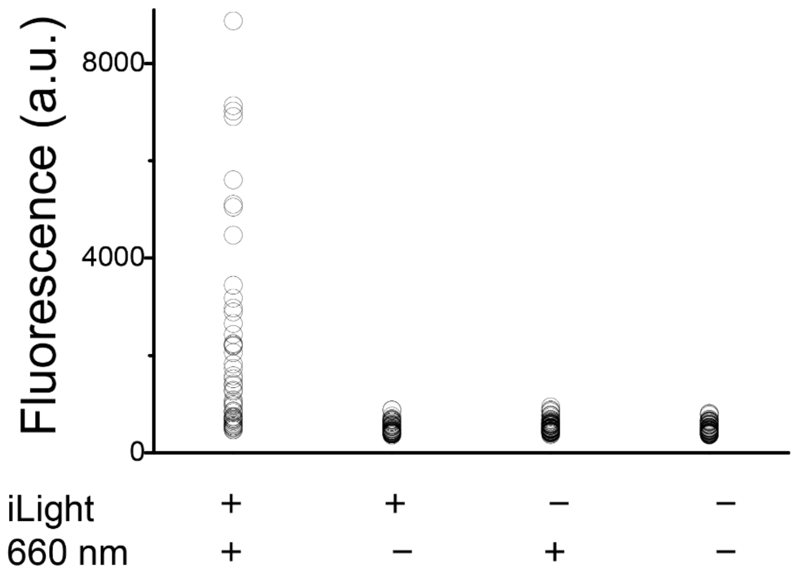

Supplementary Figure 13. Example data from a single experiment using murine hippocampal neurons co-transduced with iLight optogenetic system and mCherry reporter AAVs. mCherry reporter fluorescence intensity (in arbitrary units, a.u) in individual neurons cultured under 660 nm light or in darkness, with or without iLight is indicated as dots. Biliverdin concentration was 2 μM.

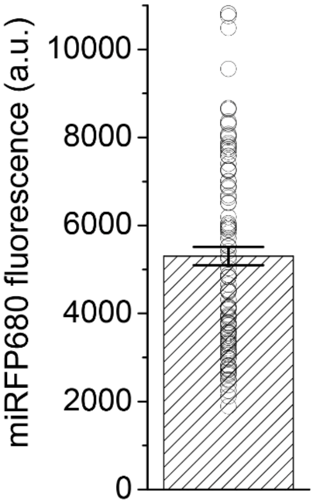

Supplementary Figure 14. Murine hippocampal neurons were transduced with AAV encoding near-infrared fluorescent protein miRFP680 at DIV7 and imaged on DIV14 (617 nm LED, 620/15 nm excitation filter, 660LP dichroic mirror, 700/50 nm emission filter). The mean fluorescence value was 5303 arbitrary units (a.u.), standard deviation 2205 a.u., n = 112 cells, coefficient of variation 42%. No biliverdin was added. Error bars indicate SEM.

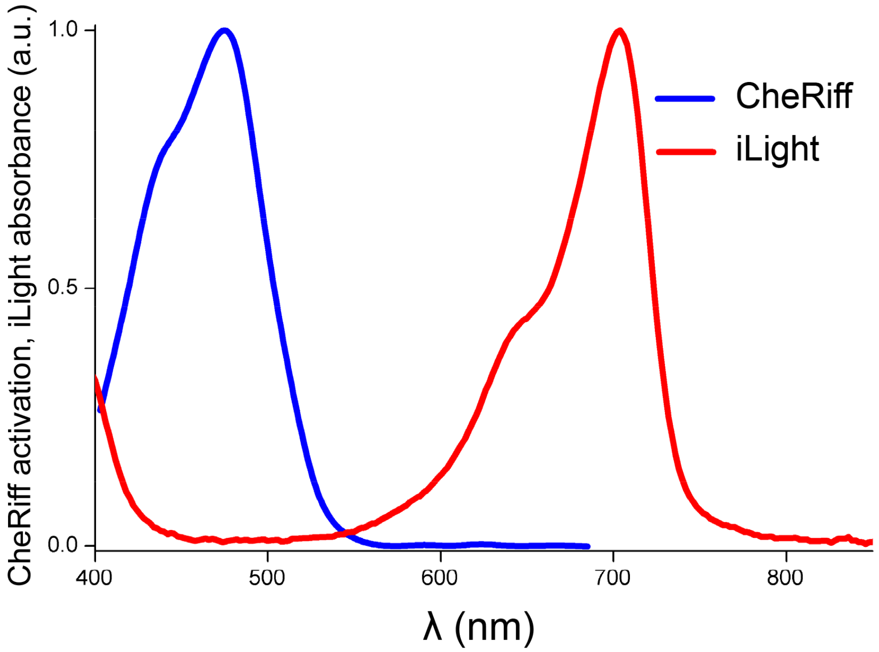

Supplementary Figure 15. Overlay of light-activation spectrum of CheRiff with absorption spectrum of iLight. The spectra were smoothed using cubic spline fit. Absorbance is shown in arbitrary units, a.u.

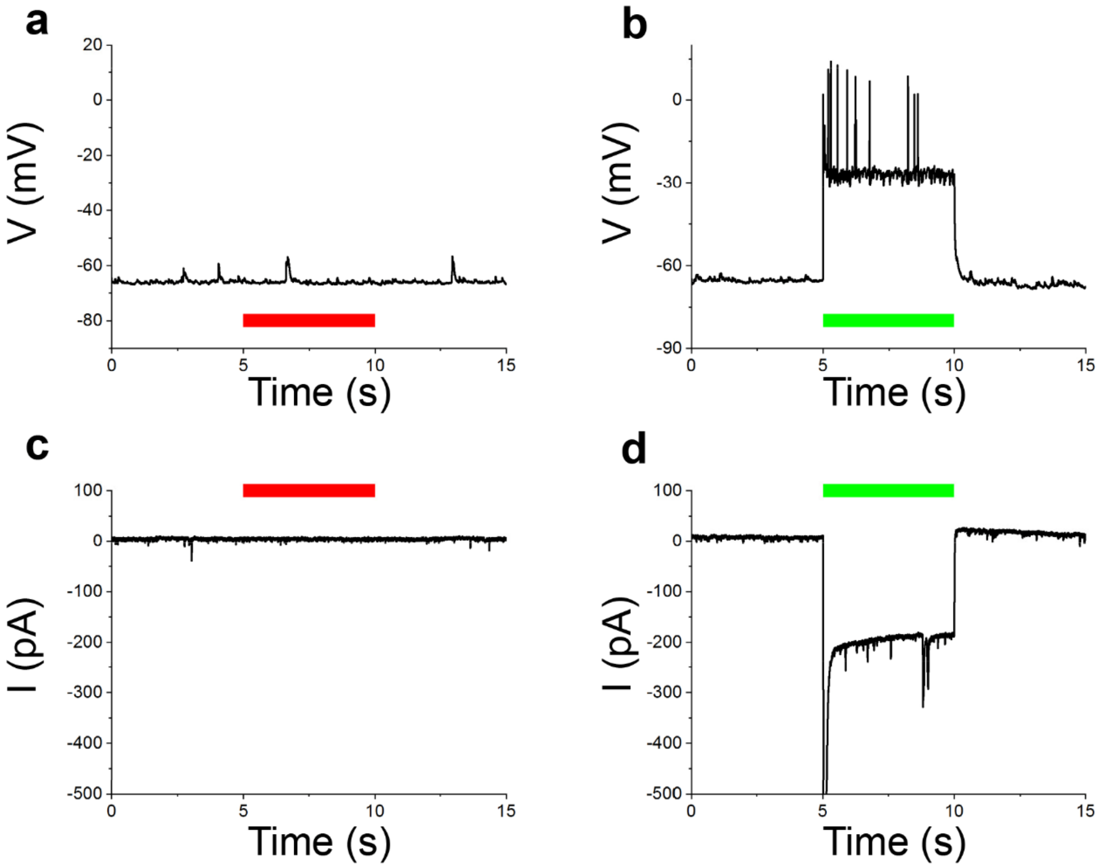

Supplementary Figure 16. Effects of near-infrared and green light on photocurrents and voltage in neurons co-expressing CheRiff and iLight. (a) Voltage trace during exposure to 656 nm LED light (red line). **(b)** Voltage trace during exposure to 505 nm light (green line). **(c)** Current trace during exposure to 656 nm light (the neuron was held at -70 mV in voltage clamp mode). **(d)** Current trace during exposure to 505 nm light (the neuron was held at -70 mV in voltage clamp mode).

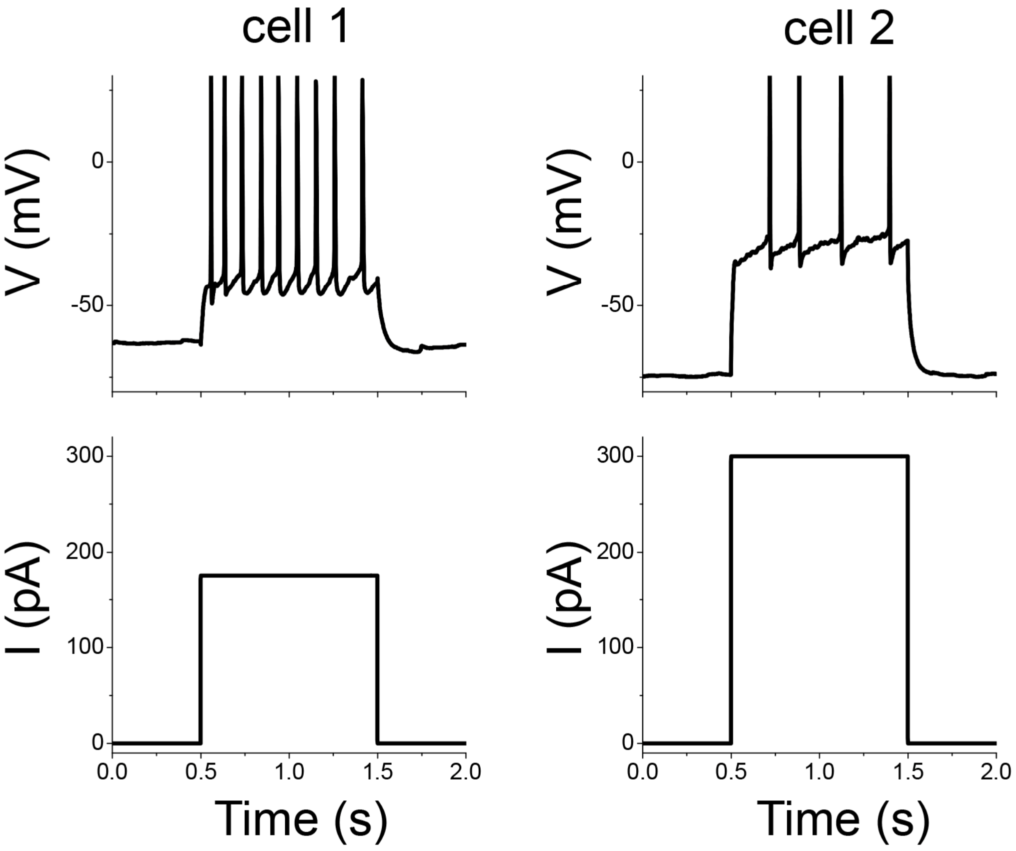

Supplementary Figure 17. Examples of action potentials fired by neurons in response to current injection. Murine hippocampal neurons transduced with iLight AAV were patch clamped at DIV14 and held in current clamp mode (zero current). Current (125 pA for cell #1 and 75 pA for cell #2) was injected through the electrode to induce action potentials.

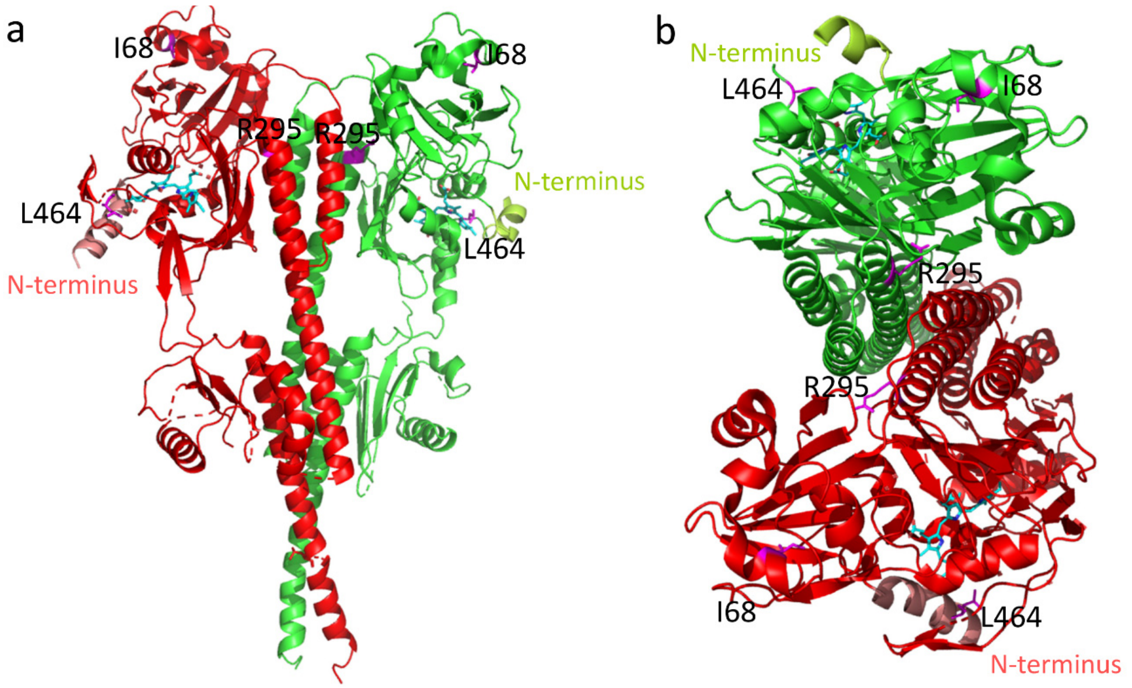

Supplementary Figure 18. Three most critical for the iLight functioning mutation mapped on the structure of the activated IsPadC-PCM dimer (PDB ID: 6ET7). (**a**) Side and (**b**) top views are shown. The three critical amino acid residues Ile68, Arg295 and Leu464, which are mutated in iLight, are shown in purple with the side chains. The biliverdin chromophore is in cyan. The activated protomer in the Pfr state is in green, and the non-activated protomer, which has remained in the Pr state after the 660 nm illumination, is in red. The N-termini of the both protomers are highlighted in the lighter hues.

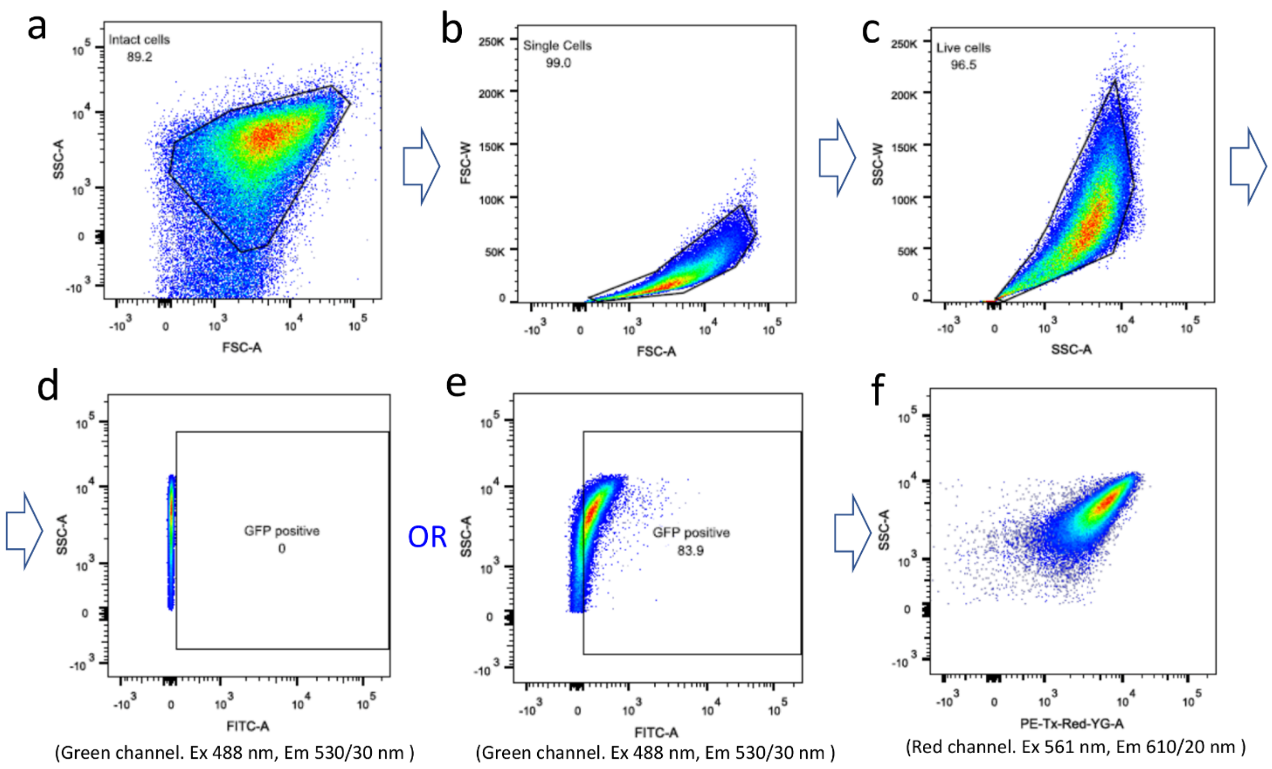

Supplementary Figure 19. Exemplifying gating strategy of FACS analysis. Bacterial cell gating was performed using three gates, for (**a**) intact cells, (**b**) single cells, and (**c**) live cells. These cells were then gated against (**d**) untransformed cells in (**e**) green (Ex. 488 nm, Em. 530/30 nm) channel for selecting msfGFP positive cells. The latter cells were used to access the brightness in green and/or (**f**) red (for mCherry: Ex. 561 nm, Em. 610/20 nm) channels and for collecting.
