# Supplementary material for: Single-component near-infrared optogenetic systems for gene transcription regulation
Source: Nat Commun. 2021 Jun 23;12:3859. doi: 10.1038/s41467-021-24212-7 (PMC8222386; doi:10.1038/s41467-021-24212-7)
Supplement: Supplementary file 2 — Reporting Summary [file 41467_2021_24212_MOESM2_ESM.pdf]

natureresearch

Corresponding author(s): Vladislav Verkhusha

Last updated by author(s): Apr 5, 2021

Reporting Summary

Nature Research wishes to improve the reproducibility of the work that we publish. This form provides structure for consistency and transparency in reporting. For further information on Nature Research policies, see Authors & Referees and the Editorial Policy Checklist .

Statistics

For all statistical analyses, confirm that the following items are present in the figure legend, table legend, main text, or Methods section.

n/a	Confirmed	
☐	☒	The exact sample size (*n*) for each experimental group/condition, given as a discrete number and unit of measurement
☐	☒	A statement on whether measurements were taken from distinct samples or whether the same sample was measured repeatedly
☐	☒	The statistical test(s) used AND whether they are one- or two-sided<br>*Only common tests should be described solely by name; describe more complex techniques in the Methods section.*
☒	☐	A description of all covariates tested
☒	☐	A description of any assumptions or corrections, such as tests of normality and adjustment for multiple comparisons
☐	☒	A full description of the statistical parameters including central tendency (e.g. means) or other basic estimates (e.g. regression coefficient) AND variation (e.g. standard deviation) or associated estimates of uncertainty (e.g. confidence intervals)
☐	☒	For null hypothesis testing, the test statistic (e.g. *F*, *t*, *r*) with confidence intervals, effect sizes, degrees of freedom and *P* value noted<br>*Give P values as exact values whenever suitable.*
☒	☐	For Bayesian analysis, information on the choice of priors and Markov chain Monte Carlo settings
☒	☐	For hierarchical and complex designs, identification of the appropriate level for tests and full reporting of outcomes
☒	☐	Estimates of effect sizes (e.g. Cohen's *d*, Pearson's *r*), indicating how they were calculated

Our web collection on statistics for biologists contains articles on many of the points above.

Software and code

Policy information about availability of computer code

Data collection	Flow cytometry data were collected using BD FACSDiva v.8.0.1 (BD Biosciences) software. Fluorescence in neurons was measured on a microscope controlled by Micro-Manager 1.3 (Vale Lab, UCSF) and Matlab R2018b (MathWorks), images were acquired by HCImage (Hamamatsu). Transmembrane current and voltage were measured with CLAMP interface software v.1.4.0 (Intan Technologies).
Data analysis	The microscopy data were analyzed using ImageJ v.1.50b (NIH). The Flow cytometry data were analyzed using FCS Express V3 (De Novo Software). Images of fluorescent neurons and traces of current and voltage were processed in Matlab R2018b (MathWorks). Bioluminescence in animals was analyzed using Living Image v.3.0 software (Perkin Elmer/Caliper Life Sciences).

For manuscripts utilizing custom algorithms or software that are central to the research but not yet described in published literature, software must be made available to editors/reviewers. We strongly encourage code deposition in a community repository (e.g. GitHub). See the Nature Research guidelines for submitting code & software for further information.

Data

Policy information about availability of data

All manuscripts must include a data availability statement. This statement should provide the following information, where applicable:

- Accession codes, unique identifiers, or web links for publicly available datasets
- A list of figures that have associated raw data
- A description of any restrictions on data availability

The main data supporting the findings of this study are available within the article and its Supplementary information. The additional data are available from the corresponding author on reasonable request. The major plasmids constructed during this research, their maps and sequences are deposited to Addgene depository. The iLight nucleotide sequence is available at GenBank.

Field-specific reporting

Please select the one below that is the best fit for your research. If you are not sure, read the appropriate sections before making your selection.

☒ Life sciences ☐ Behavioural & social sciences ☐ Ecological, evolutionary & environmental sciences

For a reference copy of the document with all sections, see nature.com/documents/nr-reporting-summary-flat.pdf

Life sciences study design

All studies must disclose on these points even when the disclosure is negative.

Sample size	No sample-size calculations were performed. Unless noted in the figure legend, standard n>3 independent experiments were performed for the most cases to allow statistical analysis.
Data exclusions	No data were excluded from analysis.
Replication	All attempts at replication were successful. Usually three independent replications of the experiments were performed.
Randomization	The experiments were not randomized because of the nature of optogenetic experiments in the article.
Blinding	The investigators were not blinded because because it was not possible for optogenetic experiments in the article.

Reporting for specific materials, systems and methods

We require information from authors about some types of materials, experimental systems and methods used in many studies. Here, indicate whether each material, system or method listed is relevant to your study. If you are not sure if a list item applies to your research, read the appropriate section before selecting a response.

Materials & experimental systems

n/a	Involved in the study	
☒	☐	Antibodies
☐	☒	Eukaryotic cell lines
☒	☐	Palaeontology
☐	☒	Animals and other organisms
☒	☐	Human research participants
☒	☐	Clinical data

Methods

n/a	Involved in the study	
☒	☐	ChIP-seq
☐	☒	Flow cytometry
☒	☐	MRI-based neuroimaging

Eukaryotic cell lines

Policy information about cell lines

Cell line source(s)	HeLa (CCL-2) cell line were obtained from ATCC. AAV-293 cells were obtained from Agilent Technologies.
Authentication	No additional authentication was performed for cells purchased in ATCC. ATCC authenticates cell lines using STR analysis, according to the product specification.
Mycoplasma contamination	Cell lines were not tested for mycoplasma.
Commonly misidentified lines (See ICLAC register)	No commonly misidentified cell lines were used.

Animals and other organisms

Policy information about studies involving animals; ARRIVE guidelines recommended for reporting animal research

Laboratory animals	The Swiss Webster 2- to 3-month-old female mice (National Cancer Institute, NIH) with body weights of 22–25 g and newborn Swiss-Webster pups of either sex were used.
Wild animals	No wild animals were used in the study.
Field-collected samples	No field-collected samples were used in the study.

Ethics oversight	All animal experiments were performed in an AAALAC-approved facility using protocols approved by the Albert Einstein College of Medicine Animal Usage Committee.

Note that full information on the approval of the study protocol must also be provided in the manuscript.

Flow Cytometry

Plots

Confirm that:

- [x] The axis labels state the marker and fluorochrome used (e.g. CD4-FITC).
- [x] The axis scales are clearly visible. Include numbers along axes only for bottom left plot of group (a 'group' is an analysis of identical markers).
- [x] All plots are contour plots with outliers or pseudocolor plots.
- [x] A numerical value for number of cells or percentage (with statistics) is provided.

Methodology

Sample preparation	Prior to analysis or sorting, bacterial TOP10 cell were washed with cold phosphate-buffered saline (PBS) and diluted in PBS to OD600 of 0.005.
Instrument	BD LSRII flow cytometer and BD FACSAria cell sorter.
Software	BD FACSDiva v.8.0.1 (BD Biosciences) and FCS Express v.3 (De Novo Software).
Cell population abundance	Collected during molecular evolution fractions of bacterial cells were less than 1%. The fraction purity was further analyzed by plating bacterial cells on Petri dishes followed by fluorescence imaging.
Gating strategy	Initial gates - FSC-A/SSC-A to discriminate cells from debris; then cells were gated in FSC-W/FSC-A to discriminate single cells; then cells were gated in SSC-W/SSC-A to discriminate live cells. Resulted population were analyzed on SSC-A/GFP and SSC-A/mCherry plots to find cells expressing msfGFP and mCherry or msfGFP only. Untransformed bacterial TOP10 cells were used for selecting negative population.

- [x] Tick this box to confirm that a figure exemplifying the gating strategy is provided in the Supplementary Information.
